# True lemurs…true species - species delimitation using multiple data sources in the brown lemur complex

**DOI:** 10.1186/1471-2148-13-233

**Published:** 2013-10-26

**Authors:** Matthias Markolf, Hanitriniaina Rakotonirina, Claudia Fichtel, Phillip von Grumbkow, Markus Brameier, Peter M Kappeler

**Affiliations:** 1 Behavioral Ecology and Sociobiology Unit, German Primate Center, Göttingen, Germany; 2 Department of Historical Anthropology, University of Göttingen, Göttingen, Germany; 3 Primate Genetics Laboratory, German Primate Center, Göttingen, Germany; 4 Department of Sociobiology/Anthropology, University of Göttingen, Göttingen, Germany

**Keywords:** Species delimitation, *Eulemur*, Madagascar, Taxonomic inflation, Integrative taxonomy

## Abstract

**Background:**

Species are the fundamental units in evolutionary biology. However, defining them as evolutionary independent lineages requires integration of several independent sources of information in order to develop robust hypotheses for taxonomic classification. Here, we exemplarily propose an integrative framework for species delimitation in the “*brown lemur complex*” (BLC) of Madagascar, which consists of seven allopatric populations of the genus *Eulemur* (Primates: Lemuridae), which were sampled extensively across northern, eastern and western Madagascar to collect fecal samples for DNA extraction as well as recordings of vocalizations. Our data base was extended by including museum specimens with reliable identification and locality information for skull shape and pelage color analysis.

**Results:**

Between-group analyses of principal components revealed significant heterogeneity in skull shape, pelage color variation and loud calls across all seven populations. Furthermore, post-hoc statistical tests between pairs of populations revealed considerable discordance among different data sets for different dyads. Despite a high degree of incomplete lineage sorting among nuclear loci, significant exclusive ancestry was found for all populations, except for *E. cinereiceps,* based on one mitochondrial and three nuclear genetic loci.

**Conclusions:**

Using several independent lines of evidence, our results confirm the species status of the members of the BLC under the general lineage concept of species. More generally, the present analyses demonstrate the importance and value of integrating different kinds of data in delimiting recently evolved radiations.

## Background

Species are the fundamental units in biology [1-3]. In fact, species are the fundamental units of comparisons in all fields of biology, including anatomy, behavior, ecology, molecular biology or physiology, underlining the importance of taxonomic studies for all biological disciplines [1,4-6]. Furthermore, species are also the currency for biodiversity classification and define regions of conservation priority, so-called biological hotspots [7,8]. Despite their fundamental importance and widespread application, identifying, defining and delimiting species is still one of the most disputed and controversial tasks in evolutionary biology [9].

Dozens of species concepts have been formulated, but none of them seems to be operational for every individual taxon (see [9-13]). De Queiroz therefore proposed a definition of species that is in agreement with all modern species concepts. Under this so-called general (metapopulation) lineage concept (GLC), the conceptualization of the notion of species and the operational criteria necessary to delimit them became separated [1,14]. Instead of using a single operational criterion, such as monophyly or interbreeding, seeing species as separately evolving metapopulation lineages through time offers and highlights the importance of using multiple lines of evidence for their delimitation [15] because different criteria can come to fixation at different times during the divergence process of two populations. In fact, different criteria can lead to important biases in estimates of biodiversity, especially in macroevolutionary and conservation studies depending on species lists [7,16], and are expected to give incongruent results for the boundaries of recently evolved radiations [15,17]. However, evaluating multiple lines of evidence not only increases our capacity to detect recently diverged populations, but also can provide stronger evidence of lineage separation when different operational criteria are in concordance [18,19].

The fauna of Madagascar has enjoyed a constant increase in species numbers in recent years. Descriptions of newly discovered species from all vertebrate groups were based on various criteria for species delimitation, however [20-28]. In this context, an almost threefold increase in the number of endemic primate species (Lemuriformes) over the last three decades has been questioned by several authors [29-31]. For example, newly described lemur species have been delimited solely based on minor variation in mitochondrial DNA (summarized in [30]). Moreover, sampling per “species” was often limited to one locality encompassed by a pair of Madagascar’s larger rivers. Thus, we have limited information on intraspecific genetic variation across a species’ geographic range, so that the documented extent of mtDNA divergence might just be a result of local population structure. Other taxa have been subject to taxonomic revision without new data and were raised to species level [32] solely based on the application of the phylogenetic species concept (PSC) in favor of the the biological species concept (BSC). These taxonomic revisions, especially in the genus *Eulemur*, were based on little evidence [31], as we outline in the following.

Based on behavioral, anatomical and cytogenetic evidence, Simons and Rumpler [33] erected and defined the genus *Eulemur* by splitting the former genus *Lemur* into two taxa, one containing only *Lemur catta* and the other containing the “true lemurs”, *Eulemur coronatus, E. mongoz, E. rubriventer, E. macaco, E. fulvus fulvus, E. f. albifrons, E. f. collaris, E. f. albocollaris, E. f. rufus* and *E. f. sanfordi.* A further subspecies, *E. f. cinereiceps,* was resurrected by Groves [32] based on a drawing by Milne-Edwards from 1890. More recent investigations revealed that this taxon is identical to *E. albocollaris* and thus the older name *E. cinereiceps* was adopted for this taxon [34].

Although hybridization occurs between wild *E. f. rufus* and *E. mongoz*[35], lineage separation of *E. coronatus, E. macaco, E. mongoz* and *E. rubriventer* from each other and from the *E. fulvus* group is considered to be significant by most authors [31,36] due to frequent sympatry, smaller social units and greater phenotypic differences. The remaining *Eulemur* taxa were treated as subspecies of the common brown lemur (*Eulemur fulvus*) and grouped into the polytypic *fulvus* group [37], also referred to as the “brown lemur complex” (BLC) [38]. Species status for *E. f. albocollaris (cinereiceps)* and *E. f. collaris* was later proposed by Wyner et al. [38], although both taxa hybridize with *E. f. rufifrons*[39,40]. In fact, hybrids of *E. cinereiceps* and *E. collaris* are not able to produce fertile offspring, but both taxa can produce fertile offspring with other members of the BLC. Although a number of studies tried to resolve the phylogeny among *Eulemur* taxa using morphology [41-43], loud calls [44], hair banding patterns [45], chromosomal banding patterns [46,47] or molecular genetics [48-54], phylogenetic relationships among *Eulemur* taxa, especially among the members of the BLC remain unresolved. Nevertheless, Groves [32] elevated all members of the BLC to species status without new evidence or new data.

Groves ([32] pp. 74-75) justified his decision to split *E. fulvus* into 7 species as follows: 

*“What one can insist on is full species status for what are currently regarded as subspecies of E. fulvus. These species are not only sharply distinct externally, but they also appear to differ consistently in craniodental characters*[43]*. Two of them, collaris and albocollaris (cinereiceps*)*, have unique DNA sequences and are already acknowledged as diagnosably distinct entities*[38]*. There is no evidence of overlap in phenotypic character states among members of the group, so they qualify as species under the PSC; there is little or no evidence that they form a genetic continuum in the wild, so they also qualify under the BSC.”*

However, Tattersall & Schwartz ([43], p. 17) stated: *"…so little of that 'craniodental’ variation can be made pertinent to relationships within the group. Clearly we are dealing with a high degree of homoplasy.''* Thus, apparently homoplastic characters have been used to delimit species under the PSC. Moreover hybridization *sensu* “a genetic continuum” is not only likely between members of the BLC, but has also been suggested for *E. rufifrons* and *E. fulvus* at Betsakafandrika [55] and *E. albifrons* with either *E. fulvus along the Mananara-*Zahamena corridor or with *E. sanfordi* north of the Bemarivo [56]*.* Thus, it appears that there is more evidence that species of the BLC form a genetic continuum in the wild than not, and explicit tests of overlap in phenotypic character states are still lacking. While all taxa may be said to represent potential new species, because of remarkable phenotypic differences of males, none of them can yet be shown to have speciated [31].

Considering the poorly justified decision to split the subspecies of the BLC into seven different species, the main aim of this study was to test this taxonomic hypothesis with new data, and to critically appraise the conceptual and empirical approaches used in delineating these and other lemur species using an approach for species delimitation that covers intraspecific variation of hypothesized lineages for multiple independent data sets. With the present paper we aim to contribute to the topic of species delimitation in recently diverged populations in general, while clarifying the taxonomy of the BLC using several lines of evidence. The usefulness of each type of data for delimiting populations of the BLC can be characterized as follows:

### Genetic data

Several studies have investigated the phylogenetic relationships of the members of the *Lemuridae*[47,52] without completely resolving the relationships within the BLC. Moreover, these studies used either only mitochondrial DNA [51] or included not all taxa or only one specimen from captivity [49,54,57] in their analyses, which limits their usefulness for delimitation of natural taxa. Therefore, we analyzed one mitochondrial and three nuclear introns to infer species boundaries of natural populations, using phylo- and population genetic methods.

### Morphology

Several authors, including Groves & Eaglen [41], Tattersall & Schwartz [43] and Groves & Trueman [42], investigated cranidodental features of the *Lemuridae* without resolving relationships between members of the BLC. Later, Viguier [58] claimed that skull disparity is more controlled by geography than by phylogeny, confirming the homoplasy found in previous studies. Because sample size for taxa of the BLC was quite small in the latter study, we revisit the morphology of lemur skulls, using a geometric morphometric approach.

### Acoustic data

Vocalizations in non-human primates are predominantly innate [59] and may thus provide an additional trait for species delimitation. Loud or long distance calls represent the most distinctive calls in the vocal repertoire and are common in most primates [60]. They typically have a species-specific acoustic structure and have therefore been used to infer phylogenetic relationships [61-67]. Macedonia & Stanger [44] investigated the phylogeny of the *Lemuridae* based on loud calls which often, but not always, consist of an introducing series of short explosive elements (chucks), followed by a long lasting scream (croak). These authors found considerable variation in what they called “disturbance advertisement calls” between members of the BLC, but they lumped all of them together for practical purposes so that variation among members of the BLC remains unknown.

### Pelage coloration

Based on genetic data and pelage coloration of a single type specimen of *E. f. rufus*, this taxon was split into two species: *E. rufus* occurring north of the Tsiribihina river and *E. rufifrons* south of it [68]. There are indeed phenotypic differences in pelage coloration among the members of the BLC, but a quantitative comparison of variation within and between populations has not been conducted so far.

Using new data from the field in combination with museum specimens, we examined variation in all four traits among the members of the BLC in order to assess the validity of all species assignments as well as to evaluate the usefulness and consistency of these four data sets in delineating species.

## Results

### Acoustic data

**Figure 1:**
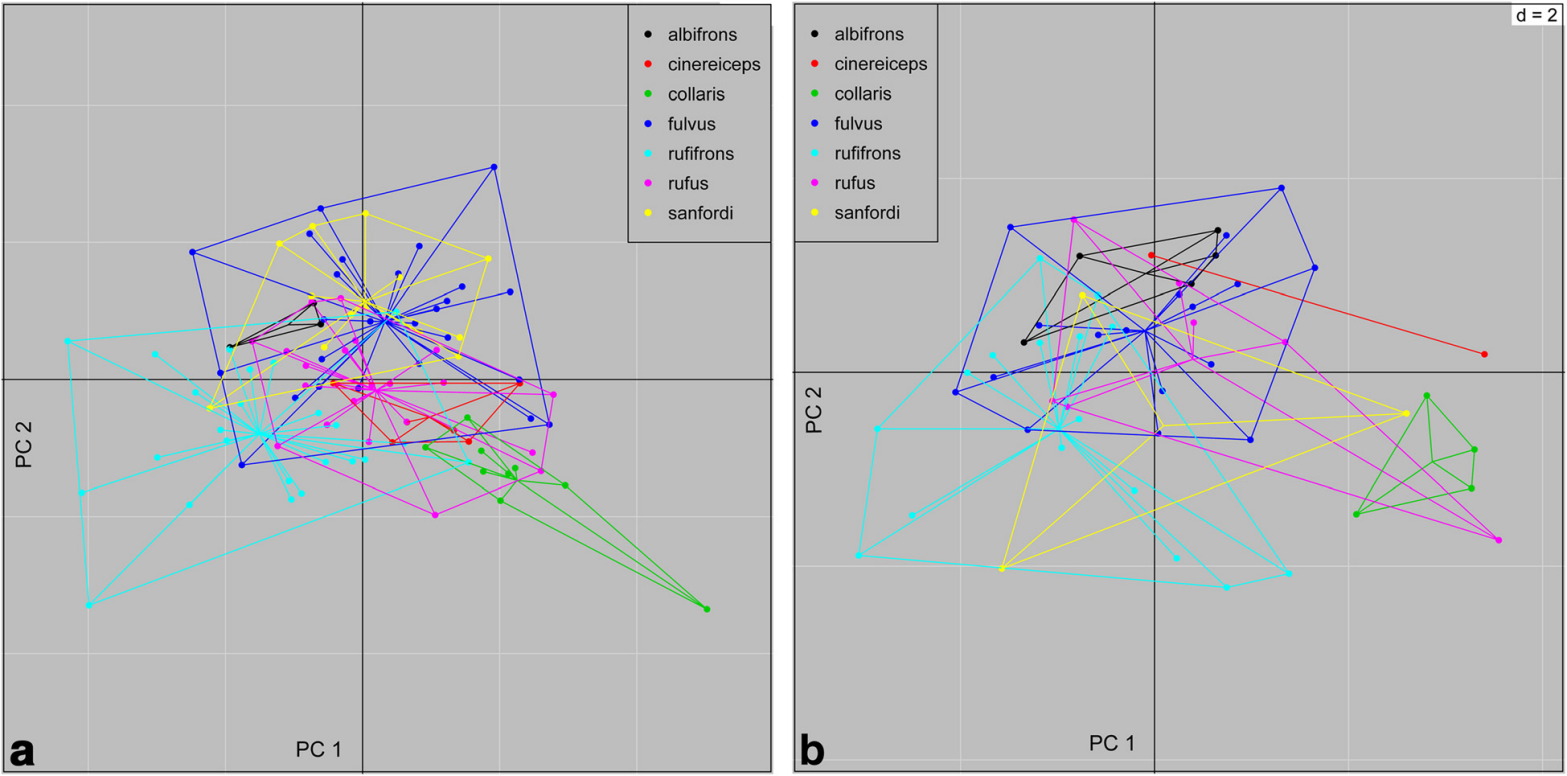
**a+b Scatter plot of bgPCA for chucks (a) and croaks (b).** Points represent individuals along the first and second principal component. A color legend for the different species is given inside the plot. p= < 0.001 (999 randomizations).

Results for the between group Principal Component Analysis (bgPCA) of chucks and croaks are depicted in Figure 1. The overall randomization test of between-group differences was significant (p< 0.001) for both call types. However, pairwise comparisons (Additional file 1: Table S6) between taxa of the permutational MANOVA (PERMANOVA) (p< 0.001) revealed only significant differences between two dyads *(E. collaris - E. fulvus* and *E. collaris- E. rufifrons)* for croaks. In contrast, chucks were significantly different between more species pairs. Whereas *E. collaris* was significantly different from all other taxa, *E. albifrons* and *E. cinereiceps* showed the fewest significant differences in pairwise comparisons. In general, the decomposition of the total variance in between-group and within-group variation revealed that only 33% of the total variation in chucks was explained by variation between taxa. Between-group variation was even lower (25%) for croaks. This pattern is well reflected by extensive overlap of groups in the scatter plots for both call types and shows that most variation in both call types is explained by intra-specific variation.

### Morphometric data

**Figure 2:**
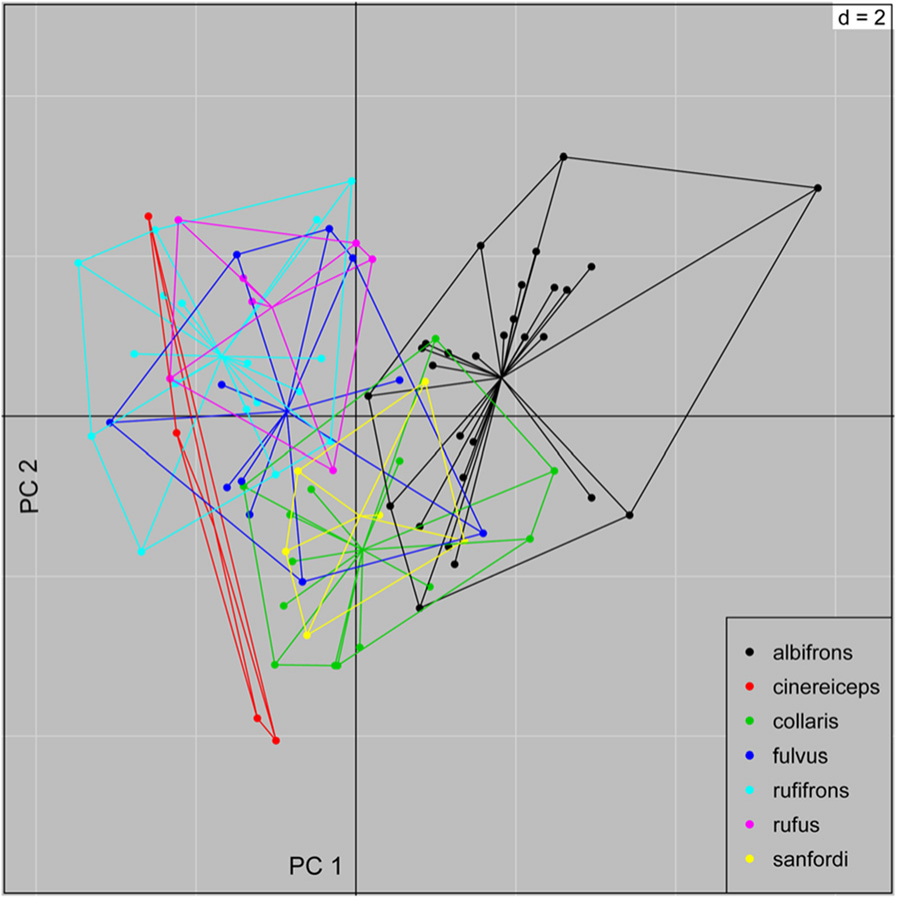
**Scatterplot of bgPCA of morphological shape analysis.** Points represent individuals along the first and second principal component. A color legend for the different species is given inside the plot. p= < 0.001 (999 randomizations).

Figure 2 shows the scatterplot of the bgPCA of procrustes shape coordinates of the members of the BLC. For comparative reasons we included also the three more distantly related taxa *E. coronatus*, *E. mongoz* and *E. rubriventer* for the morphological shape analysis (Additional file 2: Figure S4). Variance decomposition revealed that variation is much higher within (87%) than between (13%) groups. Nevertheless, the overall randomization test of between-group differences was significant (p<0.001). Results of pairwise comparisons are presented in Additional file 1: Table S7; and revealed that only half of the pairwise comparisons among members of the BLC complex showed significant differences in shape. *Eulemur cinereiceps* was only significantly different from the three smaller bodied *E. coronatus, E. mongoz* and *E. rubriventer,* but not from any of the members of the BLC. *Eulemur sanfordi* did also not differ significantly in shape from *E. albifrons, E.collaris*, *E. fulvus* and *E. rufus.* However, p-values between the geographically adjacent taxa *E. albifrons* and *E. fulvus* approached significance with p=0.068 and p=0.05, respectively. *Eulemur rufus* could not be distinguished from *E. fulvus* and *E. rufifrons* based on shape analyses. Finally*, E. coronatus, E. mongoz* and *E. rubriventer* were significantly different from each other and differed from all members of the BLC (see Additional file 2: Figure S4).

### Pelage coloration

**Figure 3:**
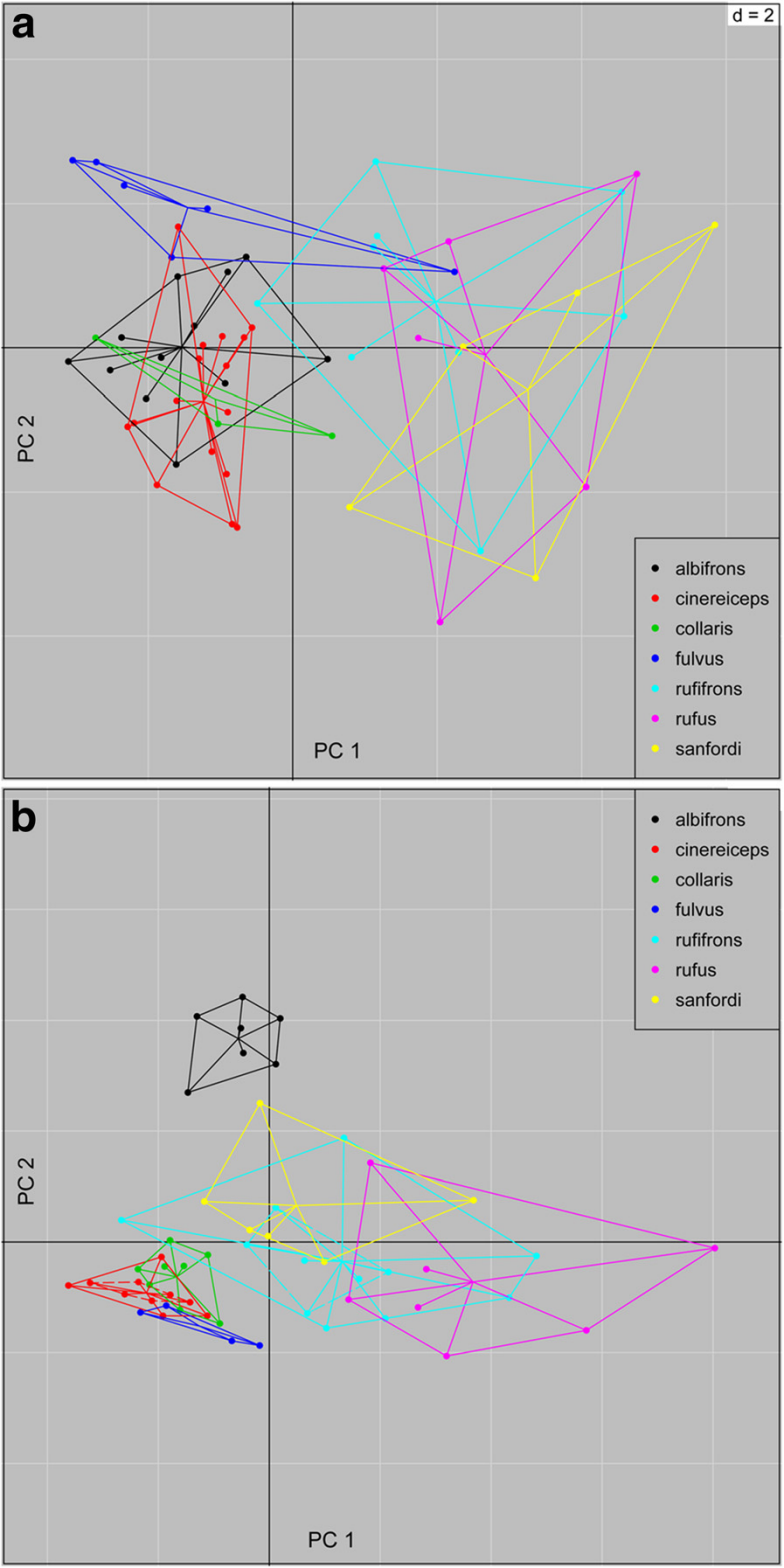
**a+b Scatterplot of bgPCA of female (a) and male (b) pelage coloration.** Points represent individuals along the first and second principal component. A color legend for the different species is given inside the plot. p= < 0.001 (999 randomizations).

Variance decomposition of the pelage coloration data revealed that in males 64% and in females 50% of the variation is explained by differences between groups. The overall test of difference between groups was significant (p<0.001). As expected from widespread sexual dichromatism, differences were more pronounced in males (Figure 3).

Subsequent pairwise comparisons significantly differentiated males of *E. albifrons* from all other taxa (Additional file 1: Table S8). Female *E. albifrons*, however, were not different from *E. cinereiceps* and *E. collaris*, but were different from the geographically adjacent *E. fulvus* and *E. sanfordi.* In contrast to Groves [68], who postulated female color differences between *E. rufus* and *E. rufifrons,* the present analysis revealed massive overlap and no significant differences between females, but between males. *Eulemur cinereiceps* was also significantly different from its neighbors, i.e. *E. collaris* and *E. rufifrons.*

### Genetic data

#### Sequence data

In total, sequence data were generated from 123 field samples. Due to high variation in the amount of genomic DNA from feces, we were unable to sequence all four loci for all individuals. Missing data are indicated in Additional file 1: Table S1. Genbank accession number are provided in Additional file 3: Table S9. The complete cytochrome B of 1140 basepairs(bp) had 57 individual haplotypes and 318 polymorphic sites. The smaller fragment of 223 bp was sequenced for additional 32 museum specimens and had 42 polymorphic sites. The number of alleles/haplotypes for the three nuclear loci were 56 for the vwf locus, 49 for the eno locus and 26 for the nramp locus, respectively (Table 1). The vwf locus had a total length of 288 bp with 56 polymorphic sites and contained two indels of one bp, one indel of 2-3 bp and one indel with seven bp. The eno-locus was 231 bp in length, contained two indels of one and two bp, one indel of three bp and had 28 polymorphic sites. The nramp- locus was 290 bp in length had 25 polymorphic sites and contained one indel. Table 1 shows the minimum, maximum and mean coverage for the individual genotyping of the three nuclear loci. Overall, there was high mean coverage of individual alleles for all loci. The AIC of JModeltest found the best fit of the cytb loci with a HKY+I+G model. The eno and vwf loci best fitted a TPM2uf+I (analyzed with GTR+I in Bayesian analysis) and a HKY+G model was favored for the nramp locus.

**Table 1:** Summary of next generation sequencing data

**NGS sequencing data**		**Coverage per individual alleles**			
**Locus**	**# of alleles**	**Mean**	**Min**	**Max**	**Indels**
vwf	56	107	10	781	4
eno	49	144	11	8678	5
nramp	26	355	22	973	1

#### Phylogenetic analyses

**Figure 4:**
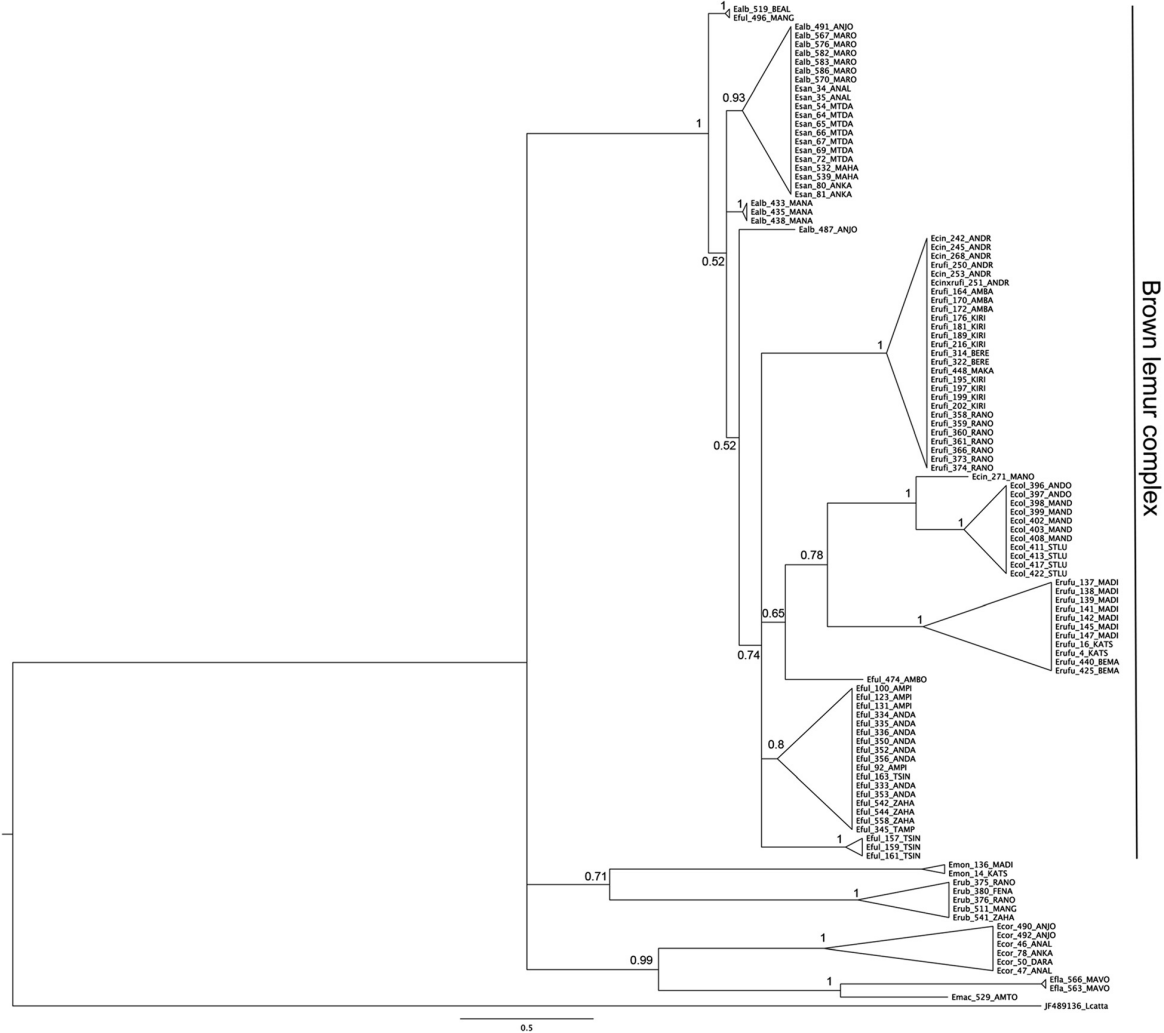
**Simplified bayesian tree of the complete cytb gene of field samples.** Labels include the designated phenotype followed by an individual identifier and an abbreviation of the sampling locality. Bayesian posterior probabilities are given at nodes. Members of the brown lemur complex are indicated by the black vertical line.

The Bayesian tree of the complete cytb is shown in Figure 4. The monophyly of the BLC is strongly supported (Bayesian PP=1.0). There was strong support for the monophyly of *E. coronatus, E. mongoz, E. macaco, E. flavifrons* and *E. rubriventer.* The relationships among clades were only poorly supported. Within the *BLC*, we found *E. rufus, E. rufifrons* and *E. collaris* to be monophyletic. *Eulemur cinereiceps, E. fulvus, E. sanfordi* and *E. albifrons* were polyphyletic. However, the individuals of *E. cinereiceps* from Andringitra are known to be hybrids [69] of *E. rufifrons* and *E. cinereiceps*.

The phylogenetic tree including museum samples revealed the same pattern as the Bayesian phylogenetic tree without museum samples. Most individuals were found in the expected clade based on their museum labels. Museum samples of *E. albifrons, E. sanfordi* and *E. fulvus* confirmed the polyphyletic pattern described above (Additional file 2: Figure S5).

**Figure 5:**
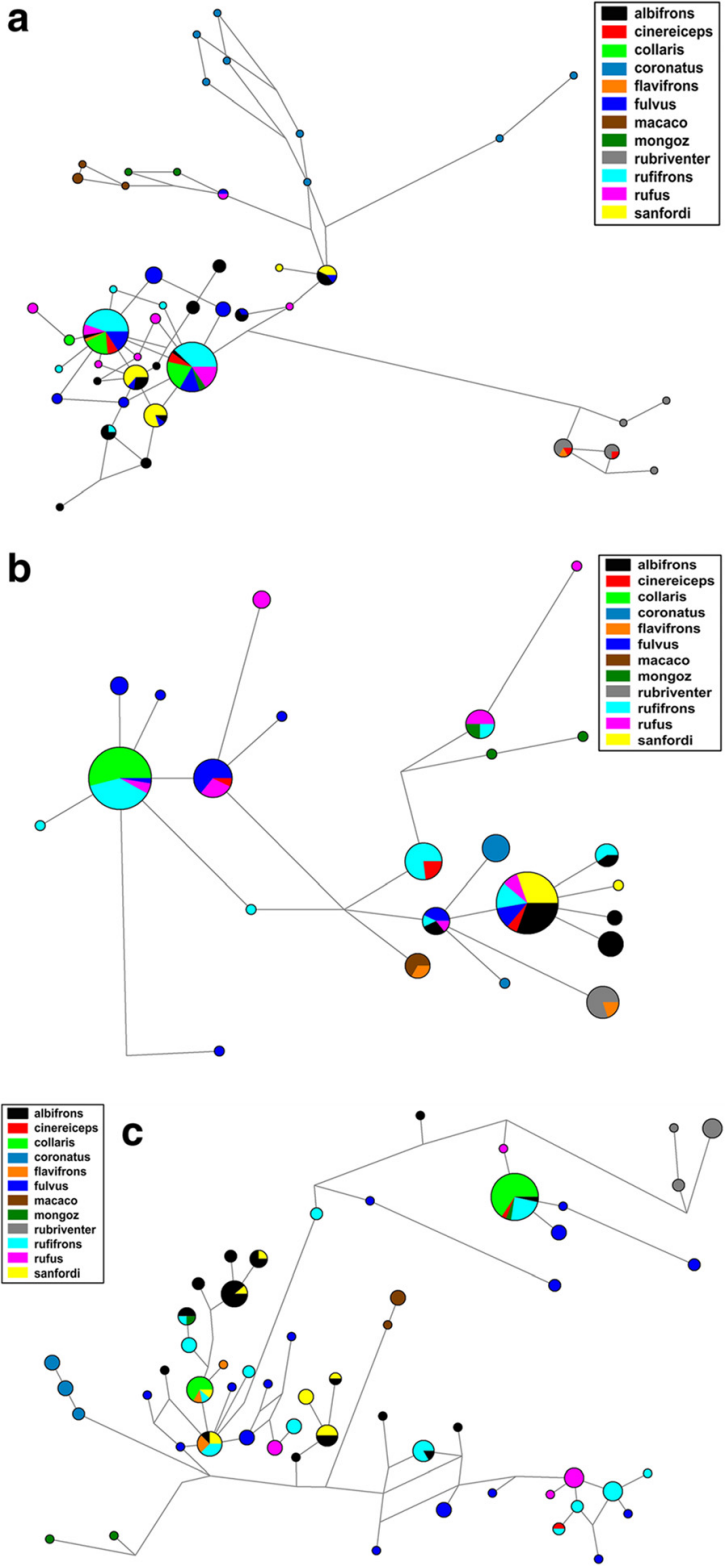
**a-c Statistical parsimony haplotype networks.** Each circle represents a different haplotype. Colors indicate the species determined after phenotype or locality. Haplotype frequency corresponds to the size of the circles and length of the branches roughly correspond to the evolutionary distance between haplotypes. **a)** eno locus, **b)** nramp locus **c)** vwf locus.

Bayesian gene trees for the three nuclear loci (Additional file 2: Figure S6a-c) showed no congruence with phylogenetic relationships revealed by the cytb locus. Although *E. coronatus, E. mongoz, E. macaco*, and *E. rubriventer* clustered together for most of the nuclear loci, phylogenetic relationships among themselves and in relation to the BLC remained unresolved. This pattern was confirmed by the statistical parsimony haplotype networks depicted in Figure 5a-c for the three nuclear loci. *Eulemur coronatus, E. mongoz, E. rubriventer* and *E. macaco* showed more species-specific distinct haplotypes and did not cluster together in the network. One individual of *E. mongoz* (27) shared haplotypes with members of the BLC. This individual was sampled in Katsepy and is a hybrid *E. mongoz* x *E. rufus* (see [35]). Some individuals labeled as *E. flavifrons* clustered within the BLC. However, we have no phenotypic information on these individuals form Manongarivo; thus they could also represent *E. fulvus.* Among the members of the BLC, we did not find any pattern corresponding to the relationships revealed by the mtDNA analyses. Several haplotypes are shared by members of different species, indicating incomplete lineage sorting for all three nuclear loci.

The genealogical sorting index showed considerable variation across loci and hypothesized lineages. Nonetheless, measures of exclusive ancestry over all loci (gsiT) were significant for all lineages except *E. cinereiceps* (Table 2.) and support lineage divergence. A gsi of 1 (= monophyly) was only estimated for several taxa for the cytb locus and for *E. mongoz* for the eno and vwf loci and for *E. rubriventer* and *E. coronatus* for the vwf locus, indicating substantial incomplete lineage sorting for our genetic loci.

**Table 2:** Genealogical sorting index (gsi) and p- values based on 10.000 permutations for the Bayesian consensus trees of all 4 loci and the combined statistic gsiT over all loci

**Species**	**gsi- cytb**	**p**	**gsi- eno**	**p**	**gsi-nramp**	**p**	**gsi- vwf**	**p**	**gsiT**	**pT**
coronatus	1,00	< 0,001	0,04	0,09	0,79	< 0,001	1,00	< 0,001	0,71	< 0,001
flavifrons	1,00	0,03	0,00	0,86	0,07	0,22	0,23	< 0,01	0,33	< 0,001
mongoz	1,00	< 0,01	1,00	< 0,001	0,50	0,01	1,00	< 0,001	0,62	< 0,001
macaco	x	x	0,01	0,69	0,24	< 0,001	0,04	0,65	0,32	< 0,001
rubriventer	1,00	< 0,001	0,69	< 0,001	0,74	< 0,001	1,00	< 0,001	0,86	< 0,001
albifrons	0,70	< 0,001	0,03	0,63	0,04	0,65	0,09	0,14	0,21	< 0,001
fulvus	0,73	< 0,001	0,12	< 0,001	0,18	< 0,001	0,18	< 0,001	0,30	< 0,001
sanfordi	0,91	< 0,001	0,51	< 0,001	0,06	0,10	0,20	< 0,001	0,42	< 0,001
cinereiceps	0,17	0,04	0,02	0,44	0,03	0,38	0,01	0,94	0,06	0,25
rufifrons	0,85	< 0,001	0,38	< 0,001	0,20	< 0,001	0,28	< 0,001	0,43	< 0,001
collaris	1,00	< 0,001	0,25	< 0,001	0,29	< 0,001	0,14	< 0,01	0,42	< 0,001
rufus	1,00	< 0,001	0,33	< 0,001	0,19	< 0,001	0,23	< 0,001	0,44	< 0,001

x = no estimate.

#### Population structure

**Figure 6:**
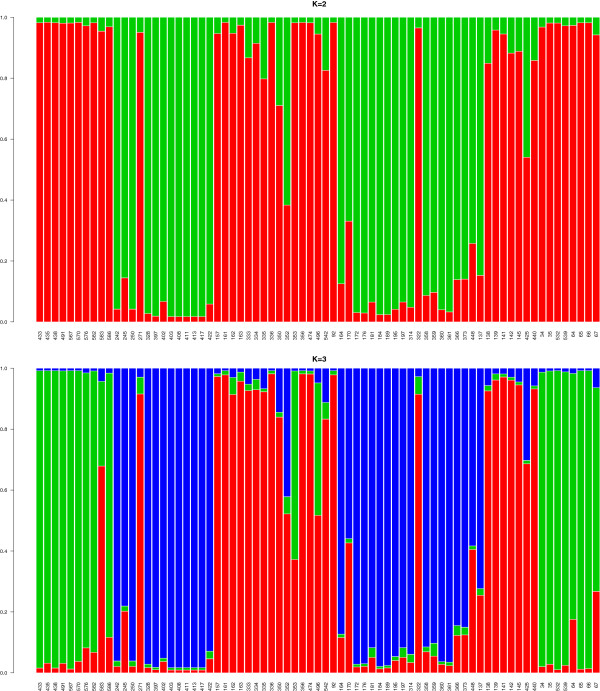
**Assignment probabilities of individual memberships to each cluster for K=2 and K=3.** The y-axes depict the assignment probabilities of each individual to one of the clusters. The x-axes show individuals in alphabetical order from left to right. *E. albifrons* =433- 586, *E. cinereiceps*= 242-271, *E. collaris*= 328-422, *E. fulvus*= 157-92, *E. rufifrons*= 164- 448, *E. rufus*= 137-440 and *E. sanfordi*= 34-67.

**Figure 7:**
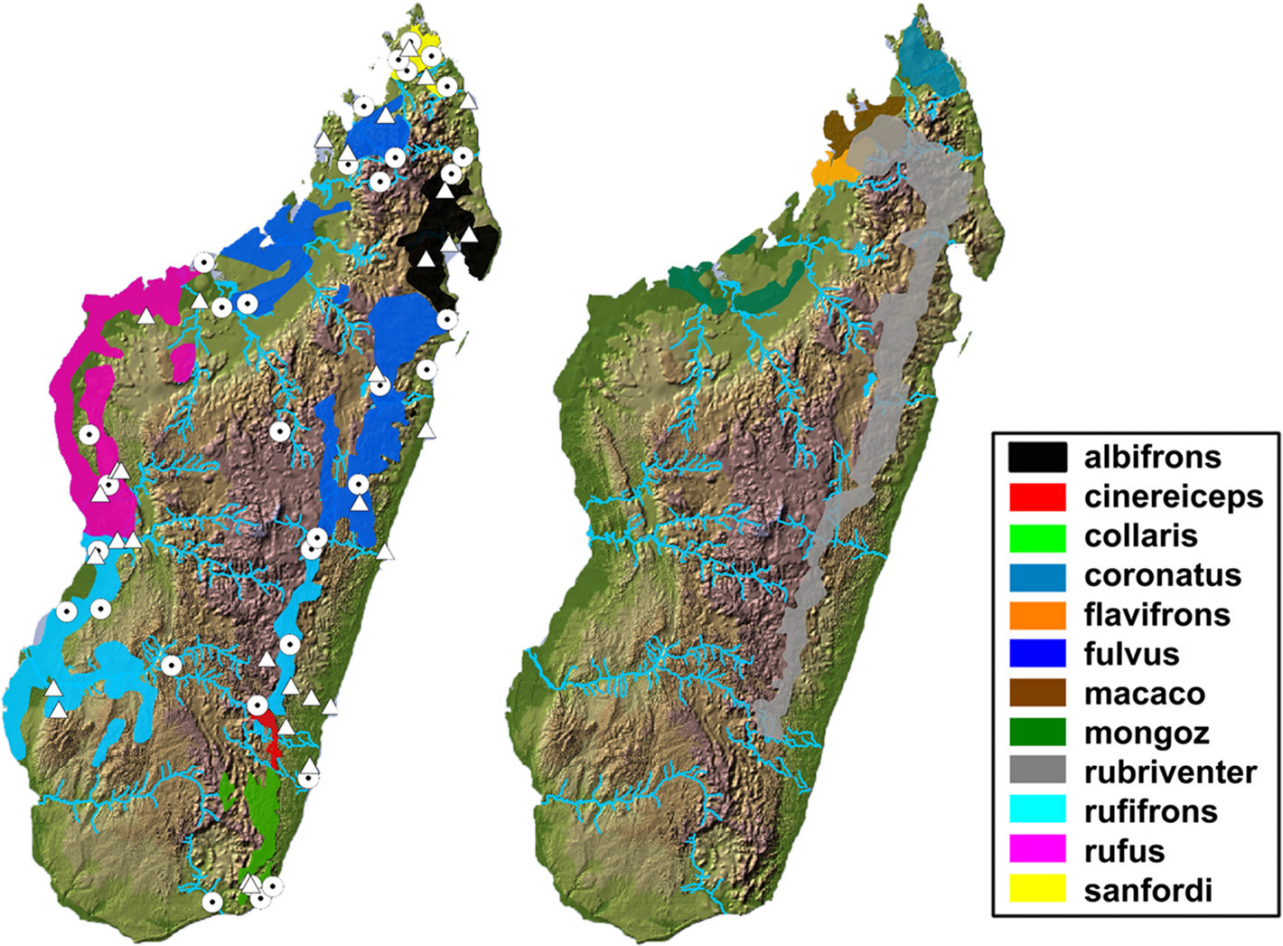
**Maps of Madagascar showing the distribution of the members of the genus *Eulemur *left= members of the *fulvus *group with our sampling localities, right= remaining members of the genus (right).** Triangles= Museum samples, circles= field samples. A color legend is shown at the right.

Bayesian population structure analysis for the members of the BLC favored a K=2 for the number of populations after the method of Evanno et al. [70] and a K=3 after the estimated ln probability of the data. Assignment plots for both K are shown in Figure 6. For K=2, with exception of individuals 271 and 322, all individuals of *E. albifrons, E. fulvus, E. rufus* and *E. sanfordi* were assigned to one cluster, and individuals of *E. cinereceps, E. collaris* and *E. rufifrons* formed a second cluster. For K=3, individuals of *E. albifrons* and *E. sanfordi* clustered together, and individuals of *E. cinereiceps, E.collaris* and *E. rufifrons* as well as individuals of *E. fulvus* and *E. rufus* showed east-west connections (see Figure 7).

**Figure 8:**
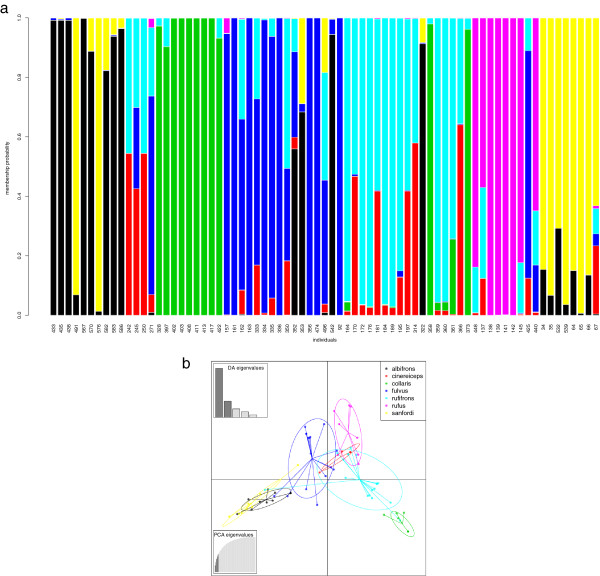
**a+b Discriminant analysis of principal components. a)** Assignment probabilities of individuals to their taxon based on 3 nuclear loci of the DAPC. The y-axis depicts the assignment probabilities of each individual. The x-axis shows individuals of taxa in alphabetical order from left to right. *E. albifrons* =433- 586, *E. cinereiceps*= 242-271, *E. collaris*= 328-422, *E. fulvus*= 157-92, *E. rufifrons*= 164- 448, *E. rufus*= 137-440 and *E. sanfordi*= 34-67.** b)** Scatterplot of DAPC with 95% confidence ellipses and number of retained principal components and discriminant functions. A color legend for both graphs is also depicted.

Results of the Discriminant Analysis of Principal Components (DAPC) on the haplotype matrix for the BLC are shown in Figure 8. The optimal alpha score suggested retention of six principal components and five discriminate functions. Most individuals could be assigned with high probability to their respective taxon. However, there was also clear evidence for a mixed nuclear genetic composition of *E. albifrons*, *E. fulvus* and *E. sanfordi*, and *E. cinereiceps, E. fulvus, E. rufus* and *E. rufifrons. Eulemur collaris* was best discriminated; *E. cinereiceps* worst. However, three out of the four *E. cinereiceps* samples were from the hybrid zone of Andringitra.

### Integration of all analyses

**Figure 9:**
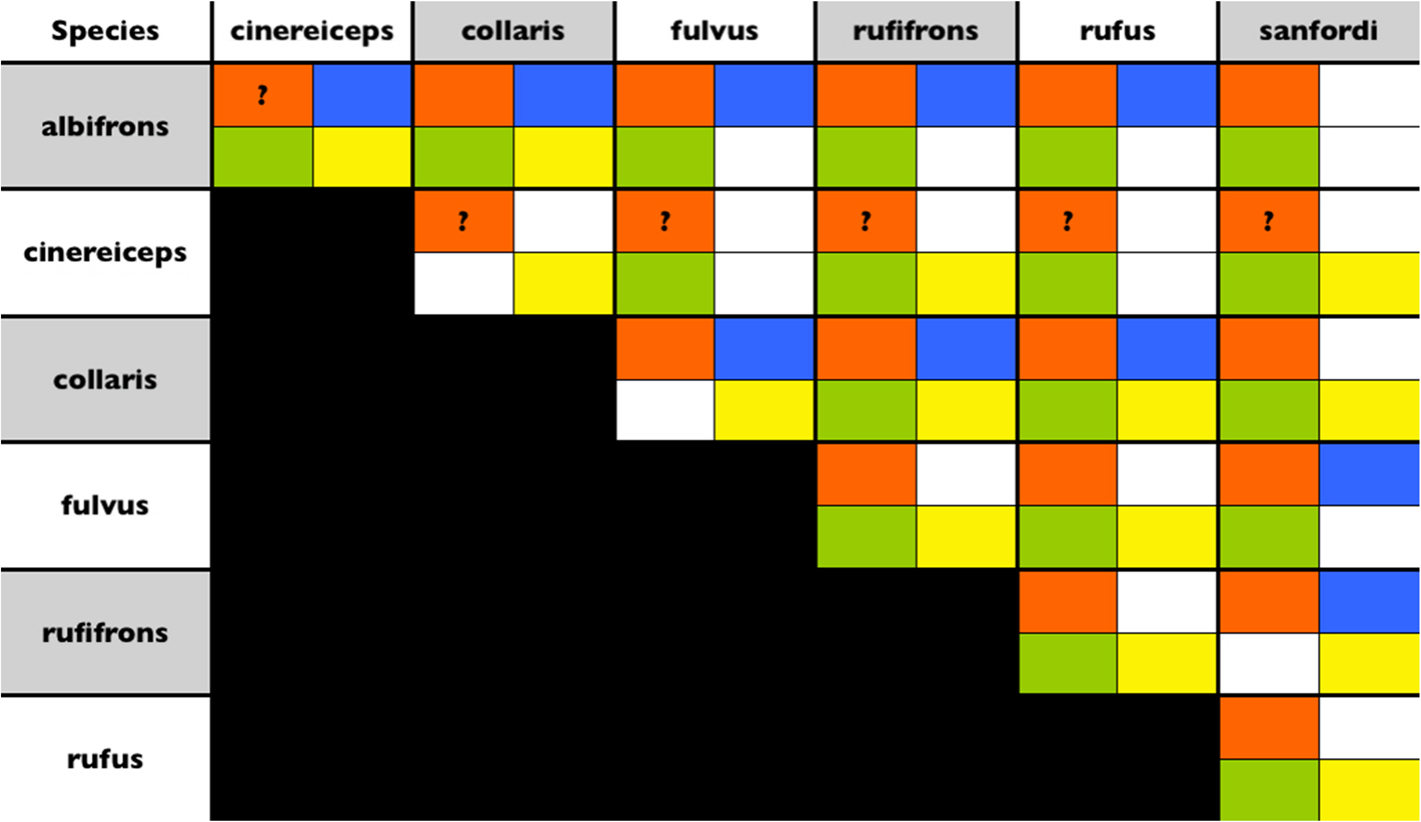
**Summary of the results of pairwise comparisons of four independent datasets.** Orange= Genetic (gsi), blue= morphology, green= pelage coloration, yellow= loud calls. Please note that we did not performed pairwise comparisons using genetic data. Therefore, we indicate significance of exclusive ancestry assessed by the gsi statistic. *E. cinereiceps* is indicated with a question mark as the gsi statistic was not significant, but this taxon was only poorly represented in our sampling and most samples were collected from the hybrid population at Andringitra.

Figure 9 summarizes the results of four different datasets and shows significant results of pairwise comparisons for morphological data, pelage coloration and acoustic parameters as well as the gsi statistic. Overall, our analyses revealed significant divergence between lineages of the BLC in all four datasets. However, the different datasets showed also considerable variation in their ability to discriminate between our predefined groups, especially in subsequent pairwise comparisons of taxa. BgPCAs of morphological shape and acoustic parameters showed that most variation in the data is explained by intraspecific variation.

For this reason, morphological shape and acoustic parameter analyses found also the smallest number of significant differences among species in pairwise comparisons. In contrast, variation in pelage coloration, especially in males, could be explained to a high degree by between-group variation, and consequently revealed significant differences between almost all species pairs. All species, except *E. cinereiceps* showed significant exclusive ancestry for the cytb locus, but also after inclusion of the three nuclear genetic loci. Monophyly of the species of the BLC for the cytb locus, however, is only evident for *E. collaris*, *E. rufus* and *E. rufifrons* (excluding the hybrids from Andringitra). Overall, results of the genetic analyses indicate a substantial amount of incomplete lineage sorting within the BLC, especially for the nuclear loci. This is shown independently by discordance among the Bayesian clustering results of STRUCTURE and the DAPC as well as in the nuclear gene trees and networks. Morphological (see Additional file 2: Figure S4) and genetic divergence of *E. coronatus, E. mongoz, E. rubriventer, E. macaco and E. flavifrons* is much more pronounced than among the members of the BLC.

As geographic and phylogenetic relationships between taxa of the BLC are crucial for a taxonomic decision, we briefly summarize results for geographically adjacent populations.

*E. albifrons* and *E. fulvus* have adjacent geographical populations at the high plateau of Tsaratanana in central northern Madagascar and along the east coast between the National Parks Mananara Nord and Zahamena (see also Figure 7). *Eulemur sanfordi* is supposed to be separated by the Maevarano du Nord river from western *E. fulvus* populations and by the Bemarivo river from southern populations of *E. albifrons.* All three can potentially meet at the headwaters of the Tasaratanana massif and/or crossing rivers. Individuals seen at Tsaratanana resemble phenotypically *E. fulvus,* but had a mixed genetic composition (ID 496). All three are significantly different in male and female coloration. Additionally, *E. albifrons* differs significantly from *E. fulvus* in shape. *Eulemur fulvus* and *E. sanfordi,* and *E. fulvus* and *E. albifrons* seem to differ also in shape, although not significantly so (p=0.05 and p=0.068). Additionally, *E. sanfordi* had high gsi values for the cytb and eno loci, suggesting independent evolution for this lineage.

*Eulemur rufifrons* is geographically adjacent to *E. rufus* in western Madagascar and to *E. fulvus* and *E. cinereiceps* in eastern Madagascar. Furthermore, *E. collaris* and *E. rufifrons* are supposed to hybridize at Berenty. Excluding hybrids from Andringitra, *E. cinereiceps* is different in mtDNA from *E. collaris* and *E. rufifrons,* and differs from both in acoustic loud calls (chucks). Differences in female and male pelage coloration of *E. cinereiceps* and *E. rufifrons* were also significant*. Eulemur collaris* and *E. rufifrons* showed significant differences in all 4 datasets and *E. rufifrons* and *E. rufus* differed significantly in pelage coloration, genetics and acoustic parameters. Finally, *E. rufus* and *E. fulvus* differed significantly in mtDNA, female and male coloration and loud calls (chucks)*.*

## Discussion

In this study, we investigated the ability of an integrative approach for the delimitation of species of a recently evolved radiation in order to falsify hypothesized lineages, in this case of the *Eulemur fulvus*[32,68]. Results clearly indicate the difficulties and discordances that can arise among and within different criteria that are frequently used to delineate taxa. Although we cannot assume that we have covered complete intraspecific variation for all taxa of this study our results also highlight the necessity for a detailed and geographically broad sampling in order to effectively compare intra- and inter- specific variation of hypothesized lineages. In the following, we discuss our results in relation to the taxonomy of the BLC, as well as the significance of the discordances among data sets and their consequences for species delineation in this and other taxonomic groups.

### How many species of true lemurs are there?

Lineage divergence occurs when populations accumulate contingent properties, such as reciprocal monophyly for different genes, distinctive ecological or morphological characters, reproductive isolation or adaptive behavioral traits [14]. As speciation is a temporal process, these different contingent properties may not begin to accumulate at the same time during the lineage separation process. In fact, different contingent properties often yield conflicting results, especially in recent or adaptive radiations [17,71]. Using different contingent properties to delimit species, however, can lead to more robust evidence of lineage separation when they are concordant [18,72]. In this study we combined multiple lines of evidence for the delimitation of seven allopatric populations of the BLC across the island of Madagascar. This evidence comprised data from mitochondrial and nuclear DNA as well as comparisons of phenotypes in skull shape, pelage coloration and call structure.

Under the general lineage concept of species, we found evidence for the lineage divergence of all seven taxa formerly considered as subspecies of *Eulemur fulvus.* These lineages seem to have diverged very recently in allopatry, probably triggered by climatic shifts during the late Pleistocene (Markolf & Kappeler, subm). As eulemurs are ecologically highly flexible and occupy most biogeographic regions of Madagascar [36], it can be assumed that genetic drift is the main mechanism generating the observed divergence of those lineages and that ecological selective processes presumably played a less important role [73]. Therefore, we cannot assume that lineages that are separated by hundreds of kilometers, such as *E. collaris* and *E. albifrons*, but occupy similar ecological niches necessarily accumulate strong differences in skull morphology or call structure (see below). Hence, it seems reasonable to make taxonomic decisions based on lineage divergence of geographically adjacent and phylogenetically closer related lineages (see also Markolf & Kappeler, subm). Following this approach, with the exception of *E. cinereiceps, E. albifrons* and *E. sanfordi,* we found evidence from three independent types of data supporting the delimitation of the taxa of the BLC as separate species.

However, *E. albifrons and E. sanfordi* were not only significantly different in male pelage coloration, but also in female coloration, a pattern not expected considering the fact that females of these two species can be hardly distinguished externally. Both species had significant gsi test statistics, indicating lineages divergence. Moreover, *E. sanfordi* had very high gsi values for the cytb and the eno loci, and DAPC could assign most *E. sanfordi* individuals with high probability to the respective cluster, suggesting exclusive ancestry for this taxon. A very recent split between these two taxa along with several past migration events (Markolf & Kappeler, subm.) seem to be responsible for a high degree of incomplete lineage sorting and less divergence in other traits analyzed here. Individual 491, treated as *E. albifrons* in our analyses, was assigned with high probability to *E. sanfordi*. In fact, we lack phenotypic information for this sample, and it may well represent *E. sanfordi* as it was sampled north of the Bemarivo. Unfortunately, security issues did not allow us to sample the area north of the Bemarivo more extensively. Thus, it remains unresolved whether *E. sanfordi* is distributed south up to the Bemarivo river, but species status is warranted. At least the museum sample from Vohemar clusters with *E. sanfordi,* indicating that this taxon had a much larger distribution than assumed today.

A clear taxonomic decision based on our data for *E. cinereiceps* is difficult. The sample from Manombo (271) clustered as a sister group to *E. collaris* in the mtDNA gene tree. The rest of our samples were collected from the hybrid population of Andringitra [69] and had mitochondrial haplotypes introgressed from *E. rufifrons.* Thus, genetically we have only one sample of “pure” *E. cinereiceps* from one locality and demarcation of this taxon based on genetics is difficult. Additionally, sample size was also very small for the museum samples and could be one explanation why *E. cinereiceps* was not found to be significantly different from any of the other members of the BLC in skull shape. However, *E. cinereiceps* differed in the acoustic structure of their chucks from adjacent *E. rufifrons* and *E. collaris,* and from *E. rufifrons* additionally in pelage coloration. Furthermore, *E. cinereiceps* and *E. collaris* have different chromosome numbers. They can therefore not produce fertile offspring [74] and would consequently qualify as species under the BSC. Further genetic investigations of the hybrid zone at Andringitra, which might shed additional light on the pattern of lineage divergence of *E. cinereiceps* in relation to *E. rufifrons* are under way (Johnson, pers. comm.).

### Discordance among data sets

We found considerable differences in the ability of different datasets to delimit among members of the BLC. None of the four data sets alone could provide enough evidence for lineage separation of all species. Genetic analyses and pelage coloration could discriminate between most members of the BLC, followed by morphological shape analysis and acoustic analyses of loud calls.

The weak discriminatory ability and low interspecific variation of the acoustic data set might be due to the structure of the calls. Most studies that used acoustic signals for species discrimination in primates analyzed calls with several syllables or even songs [63,64,75]. Those signals show necessarily more variation due to the inherent structure of the call. Furthermore, as allopatric populations normally never meet, selective pressure on calls, even those used during intergroup encounters, is probably very low. In fact, acoustic group distances and genetic group distances estimated for the cytb (data not shown) were positively correlated, indicating that genetic drift might be mostly responsible for the small divergence in acoustic parameters. Furthermore, loud calls of *E. coronatus* and *E. rubriventer* are much more different from loud calls of the members of the BLC (data not shown), suggesting that in sympatric species selective pressure on call diversity is higher than in allopatric species. Future studies in areas of overlap should employ playbackexperiments to explore this topic in more depth.

The same can be assumed for the divergence of morphological shape, as allopatric populations occupy similar ecological niches. The large overlap of the members of the BLC in the bgPCA including the three smaller eulemurs (Additional file 2: Figure S4) confirms the extensive homoplasy found in previous studies [43,58].

It can be argued that variation in pelage coloration might be influenced by environmental factors [76] and storing or preparation conditions of skins sampled in different museums. The same might be the case for acoustic variables that can be highly influenced by the environment and the distance to the animal during recordings [77]. To control for these potential errors, we used only mean values and those acoustic parameters that should be less influenced by the distance to the animal during recording [77]. And, prior to bgPCA, we run general linear models for both data types and included habitat (western dry forest and eastern humid forest) as well as museum for the color analysis as factor in the model. None of them had significant effects on the variables (data not shown). In general, data acquisition and analyses were conservative, and we aimed to cover as much intraspecific variation as possible. Therefore, we included only 17 landmarks for the analysis of shape that could be easily reproduced and placed on all available specimens. Because facial and ventral areas of museum skins were often in bad shape, areas for color measurements were chosen only on the dorsal view of the skins in order to avoid non-homologous placement of the measurement area and to cover variation of as many specimen as possible. Hence, color differences of males are definitely underestimated. As such, however, the method can be easily reproduced by other researchers even for different species.

One obvious drawback of our approach is that all four kinds of data could not be collected for the same individuals. Therefore, direct comparison or even combined analysis of morphological and genetic data such as offered in the software Geneland [78] could not be conducted. On the other hand we showed that species delimitation using several kinds of data is possible even with a completely non- invasive sampling. Especially the amount of samples for genetic analyses could not have been collected with an invasive approach.

To the best of our knowledge, this is the first study that uses Next Generation Sequencing Technology to sequence multiple independent genetic loci from feces to infer species boundaries in endangered or critically endangered primates. Following the conservative approach above, we intentionally used a high threshold to sort out potential genotyping errors. Under the assumption that sequence variants with errors occur less frequently in the dataset than sequence variants without errors, and that false alleles occur less frequently in individuals than true alleles [79], our filtering approach and a mean coverage per allele per individual ranging from 107- 355 among the three loci is unlikely to have produced false genotypes. In fact, after discarding sequence reads without both MIDs and unmatched target primers, most sequences were already filtered out. Among the remaining sequences most sequence errors turned out to be chimeras of the two most abundant sequences for an individual. Finally, that the nuclear dataset is unlikely to be influenced by genotyping errors is simply evident because of biological reasons. Although members of the BLC show a substantial mixed nuclear composition, the remaining *Eulemur* taxa have distinct haplotypes. This pattern was not necessarily expected, but confirms phylogenetic results of previous studies [51,54] and underlines the validity of our genotyping results.

Although we had known hybrids in the data and these species can hybridize in the wild and in captivity, the mixed nuclear composition of members of the BLC is more likely a consequence of incomplete lineage sorting. With the exception of the individuals from the Andringitra hybrid zone there is no indication of any geographic locality with more admixed individuals as would be expected, if hybridization was the primary cause for admixed ancestry [80]. Nevertheless, the structure results of K=3 revealed mixed ancestry for *E. albifrons-E. sanfordi, E. collaris-E. cinereiceps-E. rufifrons* and *E. fulvus-E. rufus.* However, whether this pattern is due to incomplete lineage sorting among phylogenetically closer related species or ongoing gene flow is beyond the scope of this article (but see, Markolf & Kappeler, subm).

### Delimiting species with multiple data sources

Using multiple lines of evidence, we showed that delimitation of members of recent radiations can be particularly challenging. Because different datasets can come to different conclusions about the status of species, the use of several independent data is highly recommended in order to avoid false positives. Because taxonomic classification can be treated as a hypothesis that can be modified as new evidence accumulates [81], several independent data sets allow much stronger tests of a given hypothesis.

Species delimitation in lemurs, however, has been recently criticized for relying too strongly on evidence from mtDNA alone or for using different secondary species concepts (*sensu*[14]) [30,31]. It is obvious that species delimitation based on pelage coloration or morphology alone will not be very promising in cryptic species. Nevertheless, there are other methods one could think of to falsify taxonomic hypothesis in cryptic species. Although not intended to clarify species boundaries, delBarco-Trillo et al. [82] recently showed that chemical composition in scent marks between some eulemurs are significantly different from each other. Integrating this approach into the methods for species delimitation in lemurs would be particularly useful for many of the cryptic species, as scent marks may play a role in species recognition [83]. The same applies to visual and acoustic signals, whose meaning and function to the animals in this context can be tested experimentally (e.g. [84]).

Lemurs are not the only group of mammals that has been subjected to a substantial increase in species numbers. The number of primates in general more than tripled during the last two decades [31]. In fact, the order primates has been completely revisited following the PSC [32], resulting of the elevation of many taxa from subspecies to species level without new data. A similar trend can be observed in many other mammalian orders [7,85], where similar biases have been introduced by the use of the PSC, as e.g. in ungulates [86]. Although a discussion of species concepts is way beyond the scope of this article, the PSC, which was also used to give species status to the members of the BLC, has several shortcomings that make its application inappropriate for theoretical and practical reasons. Although there are many versions of the PSC, they all emphasize a common descent, mostly referred to as monophyly, in conjunction with diagnosability, such as “A species is the smallest diagnosable cluster of individual organisms within which there is a parental pattern of ancestry and descent” [87]. Diagnosability, however, can be achieved even for the smallest possible units that might well represent demes, populations or even family groups due to limited dispersal and reproduction among geographically close individuals of the same species [88]. Therefore, the PSC is very prone to overestimating species diversity based on local genetic structure, as has recently been demonstrated with genetic data from wild mouse lemurs [30]. Cracraft [89], for example, applied the PSC and proposed species status for the Sumatran tiger based on three diagnostic characters of the cytochrome b unique to tigers from Sumatra and different from all tigers from the mainland. Our three samples of *E. rufifrons* from Ambadira have three sites diagnosably distinct from sequences of the cytochrome b of *E. rufifrons* ~20 km to the south along continuous forest. Do they qualify as distinct species? They could under the PSC, but they definitively do not, if we consider that haplotypes of the cytb are shared among individuals from Kirindy and Ranomafana, which is more than 200 km apart and separated by Madagascar’s deforested central highlands.

As evolution below and at the species level is shaped by population-level processes, taxonomic decisions require sample sizes that cover the whole intraspecific variation [85]. Furthermore, it has been shown repeatedly that gene trees (although this does also apply for trees build form other kinds of data) can substantially differ from the species tree [90-92]. Considering this and the fact that evolution at the species level is often reticulate, monophyly, especially of single genes, is in general not a good criterion for species delimitation. Using multiple genes to estimate phylogenies and delimit species is becoming popular due to advances in sequencing technology, and several new coalescent-based methods for species delimitation have recently been developed [1,93,94]. These methods seem very promising for reliably identifying recently diverged lineages. However, any deviation from the standard coalescent model (e.g. panmixia, no gene flow) is likely to overestimate species diversity, and these methods should therefore also be complemented with standard methods from morphology, ecology or behavior [89].

As conservation organizations and national governments are relying strongly on the decisions of taxonomists to assess the value of protected areas or the allocation of resources for conservation, describing and raising species based on insufficient data can also be a waste of resources and additionally lead to false decisions concerning captive or natural breeding for conservation [85].

## Conclusions

We conclude that according to the criteria investigated in this study members of the brown lemur complex (formerly *Eulemur fulvus* ssp.) are at present best classified as species according to the general lineage concept of species. As different contingent properties can arise at different times during the lineage separation process and potentially lead to ambiguous conclusions, we suggest, independent of the species concept, the utility of several independent lines of evidence, coupled with field sampling that covers intraspecific variation of the taxa under study for the delimitation of species.

## Methods

We collected data from 34 different field sites in Madagascar (Figure 7). Sampling localities were *a priori* chosen based on published distribution data of *Eulemur* species. We sampled at least 3 different populations per target taxon to cover intraspecific variation, except for *E. cinereiceps*. Additional data were collected in 5 national history museums (Additional file 1: Table S1-S3) to further increase sample size for genetic (mtDNA) analyses, and to obtain measurements on skull morphometry and fur coloration. Only museum specimens that could unequivocally be assigned to a taxon based on their phenotype, genetic characteristics or confirmed locality were included in the analyses.

### Acoustic data

In total, we analyzed 1170 loud calls from 24 *Eulemur* populations. Loud-calls were elicited by presenting species-specific loud calls given during group encounters via a loudspeaker (Davidactive, Visonik) and a Marantz digital solid state recorder (PMD 660; sampling rate: 44.1 kHz, 16 bit amplitude resolution) hidden in the vegetation. Vocalizations were recorded with a Marantz and a Sennheiser directional microphone (K6 power module and ME66 recording head with MZW66 pro windscreen; Sennheiser, Wedemark, Germany). Vocalizations were digitized using AVISOFT-SASLab pro 5.0.07 (R. Specht, Berlin, Germany). We visually inspected and sampled only calls of good quality and low background noise at a sampling frequency of 44.1 kHz. As loud calls often, but not always, consist of an introducing series of short explosive elements (chucks), followed by a long lasting scream (croak), croaks and chucks were processed and analyzed separately. A spectrogram of a typical loud call is given in Additional file 2: Figure S1.

Single calls were submitted to a fast Fourier transformation (1024-pt FFT; time step: 5 ms; frequency range: 22.05 kHz; frequency resolution: 21 Hz) with AVISOFT-SASLab pro. Frequency-time spectra were analyzed with LMA 9.2, a custom software tool to extract different sets of variables from acoustic signals [95]. We focused on acoustic variables that characterize the general call structure and are comparable with acoustic variables that were measured in other studies characterizing the structure of mammalian vocalizations [96-100]. Also, we briefly describe the acoustic variables that were used for the analysis. We measured the mean duration, the mean frequency range, the mean central frequency (DFA2) and the first and second dominant frequency bands, as well as the percentage of time of the call in which the 3rd dominant frequency could be identified [95]. Acoustic variables entered in the analysis were revealed by Pearson’s correlation analysis. We excluded variables exhibiting a correlation coefficient higher than 0.8; the remaining variables were retained and entered into the analysis.

Due to high variation in the number of calls available for each individual, we used the mean for each individual for further statistical analysis. Between-group analysis of principal components (bgPCA) was used to infer and visualize separation between taxa. BgPCA allows to separate and maximize within-group and between-group variation. This is similar but superior to discriminant function analysis (DFA), because DFA needs more cases than variables to reliably discriminate between groups [101]. Significance of group separation was afterwards tested using a randomization test with 999 randomizations. BgPCA and randomization were conducted with the Ade4 package in R (r-project.org). To identify significant differences between pairs of, we conducted a permutational MANOVA (PERMANOVA) with the program PAST [102] on the first four principal components of the bgPCA. Significance levels were corrected using the false discovery rate (FDR) [103] in R.

### Morphometric data

High resolution (18 Megapixels, RAW format) digital photographs of the ventral view of skulls were taken with a Canon 7d digital camera, a Sigma lens (70-200 mm) and with help of a photographic stand. To avoid distortion, which is higher at the fringe of the lens, photos were taken with a distance of 90 cm between the work space of the photographic stand and the sensor of the camera and with a focal length of 200 mm. Skulls were placed in the centre of the image together with a ruler. Use of modeling paste and a water level assured orientation in the horizontal plane. The program tpsDIG [104] was used to place 17 homologous landmarks on the ventral view of the skull. Landmarks (Additional file 2: Figure S2) were afterwards subjected to generalized procrustes superimposition in R, using the function procGPA of the shapes package [105]. Generalized procrustes superimposition scales, centers and rotates raw coordinates to reduce size differences between objects. BgPCA and a subsequent randomization test on the superimposed coordinates were applied to decompose intra- and interspecific variation and to test for differences between species. The function testmeanshapes of the shapes package in R was used to test for pairwise difference between taxa with subsequent FDR correction of p-values.

### Fur color data

Following the method of Bergman & Beehner [106], raw digital photographs of the dorsal view of museum skins were taken with the same equipment as mentioned above. Pictures were intentionally underexposed to avoid clipping of color channels [107]. Focal length was reduced to 70 mm, and a color chart (MiniColorChecker, Munsell) was included in each photo to control for differences in ambient light conditions. To determine color variation, each image was opened with the raw converter in Photoshop CS5 and all parameters were set to zero, except for the temperature, which was set to 5100K for all photos. Using the PictoColor plugin (http://www.pictocolor.com), we applied a new color profile to each photo based on the 24 colors of the color checker chart.

We measured three areas of each skin by taking the Red, Green and Blue value (RGB) of an area of 50 × 50 pixels with the help of the rectangular marking tool (Additional file 2: Figure S3). One area was a combined measure of two squares of 50 × 50 pixels of the dorso- lateral torso of each specimen. The second area was located on the meso-dorsal stripe that some taxa possess and the third on the centre of the head. Grids and reference lines were used to control for homologous positions of the rectangles in each specimen. Mean RGB values were noted down in an Excel sheet for each area for further statistical analysis. BgPCA with subsequent permutational MANOVA on the first two principal components was conducted to test for pairwise difference between taxa.

### Genetic data

More than 500 individual fecal samples were collected from eulemurs in the field from 2008-2011. Feces were stored on silica gel and/or 90% ethanol. After completion of fieldwork, feces were stored at 4°C until DNA extraction. Genomic DNA from the fecal samples was extracted using the QIAamp DNA Stool kit DNA (Qiagen) with a slightly modified protocol as follows. Samples were run for 24 hours at room temperature on a lab rotator in ASL buffer and only a 1/2 InhibitEx tablet was used for 600 μl supernatant of ASL-Buffer. Additionally, centrifugation steps of Qiagen spin columns were done at 8000 rpm instead of 13000 rpm as suggested in the Qiagen protocol. The same sample was sometimes extracted two or three times, which still resulted in sufficient amount of genomic DNA for PCR. A total of 116 individuals were finally used for the different genetic analyses (see Additional file 1: Table S1).

DNA extraction and subsequent PCR for the museum samples was done at a different institution (Abt. Historische Anthropologie, Universität Göttingen) under strict conditions for contamination prevention following Hummel [108], such as separation of pre- and post-PCR laboratories and the use of disposable protective clothing, glasses, and disposable gloves. Further, all experiments took place with disposable laboratory ware, such as pipette tips and cups, while workbenches and other laboratory equipment were cleaned with detergents (AlconoxTM Detergent, Aldrich, Germany), bi-distillated water and ethanol before use for each sample. Automatic DNA extraction of these samples was done with the QIAGEN EZ1 robotic station and the QIAGEN EZ1 DNA Tissue Kit.

Whereas the whole (1140 bp) cytochrome B gene was analyzed for fecal samples, only a shorter fragment of 223 bp was analyzed for the museum samples consisting of tissue remnants on skulls or pieces of the skin. Primers, PCR mixtures and annealing temperatures are listed in Additional file 1: Tables S4 and S5. We used Roche High Fidelity Taq Polymerase for amplification of DNA extracted from feces and the Qiagen Multiplex PCR plus Kit for the extractions of ancient DNA from museums.

#### Nuclear DNA

Three nuclear introns were sequenced, using 454 amplicon sequencing on a Roche GS Junior 454 Sequencing platform, which allows to directly score both alleles in a diploid individual without extensive cloning procedures. However, prior to sequencing, amplicon libraries have to be constructed and each amplicon requires its own combination of MID tags to assign individuals to the correct sequence after pooling all amplicons for emulsionPCR and subsequent sequencing. A two-step PCR procedure was used to construct amplicon libraries of the three introns nramp (natural resistance macrophage protein), vwf (van willebrand factor) and eno (enolase). Initially, target-specific primers (Additional file 1: Table S4) were designed with help of published sequences from Horvath et al. [49] and Perelman et al. [57]. These primers were equipped with a universal tail (M13) for the first PCR. After control on an agarose gel, PCR products were purified using magnetic beads (Beckmann and Coulter), and purified products were diluted with Molecular Biology Grade Water to approximately equimolar (5-20 ng/μl) concentrations for the next PCR. Primers for the second PCR included the GS Junior Titanium fusion primer sequences, 1-10 different MIDs for both forward and reverse primers and the template-specific sequence, which in our case were the universal tails of the previous PCR. This approach allowed us to use only 10 different forward and reverse fusion primers to individually tag 10×10=100 individuals for all three introns. The second PCR was run with the same conditions as the first. For the rest of the procedure we followed the GS junior Amplicon Library Preparation Method Manual, the GS Junior emPCR Amplification Method Manual Lib-A and the GS Junior Sequencing Method Manual from Roche.

#### Genotyping of individuals

After initial quality filtering and processing (i.e. adaptor removal) by the Roche/454 GS Junior software, further preprocessing was carried out by custom Perl scripts. First, sufficiently long reads were selected that perfectly matched a pair of barcode (MID) tags. Target-specific primers were removed that need to be found at the 5′ and (as reverse complement) at the 3′ end. All reads from the same gene locus were moved to a separate file. Then each sequence file was compressed by (a) removing (duplicate) reads with a perfectly identical copy in the same individual, and (b) noting the number of read copies in the FASTA comment, together with the individual identifier (corresponding to the MID tag pair).

After preprocessing, the unique sequences were aligned in SeaView [109], using the muscle alignment option and subsequent manual inspection for each intron separately. Sequences were sorted by individual in Geneious 4.5 (Biomatters). As 454 sequencing is prone to sequencing errors, specifically chimeras and insertion/deletion errors due to homopolymers [110], we used the following protocol to infer the correct genotypes from all variants:

–All sequences with <10-fold coverage were discarded from the dataset.

–Insertions/deletions that occurred only in one non-duplicate sequence in the whole dataset of a gene locus were discarded from the dataset, because they were likely to be a consequence of homopolymers.

–Variants of each individual were sorted for coverage and checked for chimeras. If one of the sequences was likely to be a chimera of the sequences with highest coverage, they were discarded.

–The two sequences with highest coverage were finally taken as the true alleles for diploid individuals, if more than one sequence was left in the end.

#### Phylogenetic analyses

Final alignments for each locus were produced with SeaView and manually inspected by eye. The best fitting substitution models were calculated for each locus with jModeltest2 [111] and chosen based on Akaike’s Information Criterion (AIC). Haplotypes were collapsed using FaBox [112] and translated into a genotype matrix for population genetics analyses. Input files for different software packages were also created with help of the web server GALAXY [113] and Microsoft Excel.

For the combined analysis of the cytb of museum and field samples, a simple Neighbor Joining Tree was calculated using the pairwise deletion option in SeaView with 10.000 bootstraps. Phylogenetic trees for the cytb without museum samples and the three nuclear loci were estimated separately using MrBayes 3.2.1. [114]. In all analyses, we used two runs with four Monte Carlo Markov Chains (MCMC), the default temperature of 0.2, 10.000.000 generations and a sampling frequency of 1000. After a burn-in of 25% we retained 15.002 trees. Substitution model parameters were adjusted as before according to the results from jModeltest. The program Tracer and the uncorrected potential scale reduction factor (PSRF, should approach one) in MrBayes were used to check for the adequacy of the burn-in and sufficient convergence of the Markov chains.

We calculated the genealogical sorting index (gsi) [115] to quantify exclusive ancestry of lineages. The gsi ranges form zero to one, where zero indicates complete lack of divergence and one indicates monophyly. As the significance of the gsi statistic is measured through randomizations of group labels across the tips in a rooted gene tree, hypothesized lineages are tested against the null hypothesis of no divergence. Therefore, significance of the gsi statistic indicates exclusive ancestry of lineages, whereas the value of the gsi measures the degree of lineage divergence. The gsi was calculated separately and combined for all loci using the Bayesian phylogenetic trees.

As phylogenetic trees are often not appropriate to illustrate relationships due to reticulate evolution or incomplete lineage sorting, we calculated statistical parsimony haplotype networks for the three nuclear loci using NETWORK 4.611 (http://www.fluxus-engineering.com) [116].

#### Population structure

We used two population genetic methods to test for population structure with the nuclear genotype matrix. STRUCTURE version 2.2 [117,118] was used for Bayesian clustering of individuals into populations. To infer the correct number of K (clusters), 20 independent runs of 1.000.000 generations and a burn-in of 250.000 generations was used in an admixture model with correlated allele frequencies from K=1- 20. The number of K was inferred over all runs with STRUCTURE HARVESTER [119] after the ln likelihood of the data and after the method of Evanno et al. [70]. CLUMPP [120] was used to permute over all runs for a given K, and assignment probabilities were plotted in R.

Discriminant Analysis of Principal Components (DAPC) [121] of the adegenet package in R was used to infer the probability of individuals belonging to predefined phenotypic species. DAPC is a multivariate method to infer the genetic structure of populations. The advantage of this method is that it does not assume Hardy Weinberg Equilibrium and linkage disequilibrium as STRUCTURE and other population genetic clustering methods, which is likely to be violated in most natural populations [120]. The alpha score was used to choose the number of retained principal components and subsequent discriminant functions in order to avoid over-fitting of the data by retaining to many principal components as suggested by the manual.

## Competing interests

The authors declare that they have no competing interests.

## Authors’ contributions

MM, CF, PMK conceived the study and wrote the manuscript. MM, CF and HR collected and analyzed data. PG and MB analyzed genetic data. All authors have read and approved the final manuscript.

## Supplementary Material

Additional file 1 **Table S1.** List of genetic samples used in this study. # = Sequence data available, NA= no sequence data available, x/y = GPS coordinates, ID= field or museum number (NHM= National History Museum, NHMB= Naturhistorisches Museum Berlin, MCZ= Museum of Comparative Zoology), POP= Population (IVOL=Parc Ivoloina, MANA=Mananara National Parc, ANJO= Anjombalava, BEAL= Bealanana, MARO=Marojejy, ANDR= Andringitra, MANO= Manombo Special Reserve, ANDO= Andohahela, MAND= Mandena, STLU= St.Luce, ANAL= Analamerana, DARA= Daraina, ANKA= Ankarana, MAVO= Manongarivo, AMPI= Ampijoroa, TSIN= Tsinjoarivo, ANDA= Andasibe, AMBO= Ambohitantely, MANG= Mangindrano, ZAHA= Zahamena, AMTO= Ambato, KATS= Katsepy, MADI= Madirovalo, RANO= Ranomafana, FENA= Fenarive Est, AMBA= Ambadira, KIRI= Kirindy, BERE= Berenty, MAKA= Massif du Makay, BEMA= Tsingy de Bemaraha, MTDA=Montagne D’Ambre, MAHA=Mahagaga). **Table S2.** Museum specimen used for morphometric analysis. AMNH= American Museum of National History, New York; USNM= Smithsonian Institution Washington D.C.; NHM= National History Museum, London; MCZ= Museum of Comparative Zoology, Boston. m= male, f= female. **Table S3.** Museum specimen used for pelage color analysis. AMNH= American Museum of National History, New York; USNM= Smithsonian Institution Washington D.C.; NHM= National History Museum, London; MCZ= Museum of Comparative Zoology, Boston. m= male, f= female. **Table S4.** Primer and annealing temperatures used in this study. MID= Multiplexidentifier, °C= Annealing temperature. **Table S5.** PCR reaction mixtures. **Table S6.** fdr- corrected p- values for pairwise comparisons after permutational MANOVA of loud calls. n.s.= not significant. **Table S7.** FDR- corrected p-values for pairwise comparison of shapes. n.s.= not significant. **Table S8.** FDR-corrected p-values for pairwise comparisons of permutational MANOVA for pelage coloration. n.s.= not significant. Click here for file

Additional file 2 **Figure S1.** Spectrogram of typical disturbance and advertisement call of members of the fulvus group. X-axes= Time in seconds, y-axes= Frequency in kHz. **Figure S2.** 17 homologous landmarks used for geometric morphometric analyses. 1= Prosthion, 2= Posteriormost point of the left incisive foramen, 3= Premaxilla- maxilla suture, 4= Meeting point of premaxilla- maxilla suture and canine, 5= Posteriormost point of canine alveolus, 6= Maxilla- palatine suture, 7=Staphilio, 8= Posterior-jugal contact of alveolar ridge and 1st molar , 9= Lateralmost point of orbitum, 10= Lateralmostpoint of jugale, 11= Medialmostpoint of the braincase, 12= Lateralmostpoint of basisphenoid- vomer suture, 13= Lateralmostpoint of basioccipitale- basisphenoid suture 14= Lateralmostpoint of the meatus acousticus externus, 15= Basion, 16= Lateralmostpoint of foramen magnum, 17= Inion. **Figure S3.** 50 x 50 pixels measured with rectangular marqee tool in Adobe Photoshop. **Figure S4.** Scatterplot of bgPCA of morphological shape analysis including *E. coronatus, E. mongoz* and *E. rubriventer.* Points represent individuals along the first and second principal component. A color legend for the different species is given inside the plot. p= < 0.001 (999 randomizations). **Figure S5.** Neighbor joiningtree of the cytb locus including museum samples. **Figure S6a-c.** Bayesian gene trees of nuclear loci. a) eno, b) nramp c) vwf. Click here for file

Additional file 3: Table S9 Comma separated file with Genbank accession numbers. Click here for file
