# Supplementary material for: True lemurs…true species - species delimitation using multiple data sources in the brown lemur complex
Source: BMC Evol Biol. 2013 Oct 26;13:233. doi: 10.1186/1471-2148-13-233 (PMC3819746; doi:10.1186/1471-2148-13-233)
Supplement: Additional file 1 — Table S1. List of genetic samples used in this study. # = Sequence data available, NA= no sequence data available, x/y = GPS coordinates, ID= field or museum number (NHM= National History Museum, NHMB= Naturhistorisches Museum Berlin, MCZ= Museum of Comparative Zoology), POP= Population (IVOL=Parc Ivoloina, MANA=Mananara National Parc, ANJO= Anjombalava, BEAL= Bealanana, MARO=Marojejy, ANDR= Andringitra, MANO= Manombo Special Reserve, ANDO= Andohahela, MAND= Mandena, STLU= St.Luce, ANAL= Analamerana, DARA= Daraina, ANKA= Ankarana, MAVO= Manongarivo, AMPI= Ampijoroa, TSIN= Tsinjoarivo, ANDA= Andasibe, AMBO= Ambohitantely, MANG= Mangindrano, ZAHA= Zahamena, AMTO= Ambato, KATS= Katsepy, MADI= Madirovalo, RANO= Ranomafana, FENA= Fenarive Est, AMBA= Ambadira, KIRI= Kirindy, BERE= Berenty, MAKA= Massif du Makay, BEMA= Tsingy de Bemaraha, MTDA=Montagne D’Ambre, MAHA=Mahagaga). Table S2. Museum specimen used for morphometric analysis. AMNH= American Museum of National History, New York; USNM= Smithsonian Institution Washington D.C.; NHM= National History Museum, London; MCZ= Museum of Comparative Zoology, Boston. m= male, f= female. Table S3. Museum specimen used for pelage color analysis. AMNH= American Museum of National History, New York; USNM= Smithsonian Institution Washington D.C.; NHM= National History Museum, London; MCZ= Museum of Comparative Zoology, Boston. m= male, f= female. Table S4. Primer and annealing temperatures used in this study. MID= Multiplexidentifier, °C= Annealing temperature. Table S5. PCR reaction mixtures. Table S6. fdr- corrected p- values for pairwise comparisons after permutational MANOVA of loud calls. n.s.= not significant. Table S7. FDR- corrected p-values for pairwise comparison of shapes. n.s.= not significant. Table S8. FDR-corrected p-values for pairwise comparisons of permutational MANOVA for pelage coloration. n.s.= not significant. [file 1471-2148-13-233-S1.pdf]

Table S1: List of genetic samples used in this study. # = Genbank accession number, x/y = GPS coordinates, ID= field or museum number (NHM= National History Museum, NHMB= Naturhistorisches Museum Berlin, MCZ= Museum of Comparative Zoology), POP= Population (IVOL=Parc Ivoloina, MANA=Mananara National Parc, ANJO= Anjombalava, BEAL= Bealanana, MARO=Marojejy, ANDR= Andringitra, MANO= Manombo Special Reserve, ANDO= Andohahela, MAND= Mandena, STLU= St.Luce, ANAL= Analamerana, DARA= Daraina, ANKA= Ankarana, MAVO= Manongarivo, AMPI= Ampijoroa, TSIN= Tsinjoarivo, ANDA= Andasibe, AMBO= Ambohitantely, MANG= Mangindrano, ZAHA= Zahamena, AMTO= Ambato, KATS= Katsepy, MADI= Madirovalo, RANO= Ranomafana, FENA= Fenarive Est, AMBA= Ambadira, KIRI= Kirindy, BERE= Berenty, MAKA= Massif du Makay, BEMA= Tsingy de Bemaraha, MTDA=Montagne D'Ambre, MAHA=Mahagaga), #= Genebank Accession Number

ID	Pheno-type	Species	POP	eno	nramp	vwf	cytb	x	y	comment
384	Ealb	hybrid	IVOL	#	#	NA	NA	S18 03.405	E49 21.599	removed
389	Ealb	hybrid	IVOL	#	#	#	NA	S18 03.405	E49 21.599	removed
390	Ealb	hybrid	IVOL	#	#	#	NA	S18 03.405	E49 21.599	removed
433	Ealb	albifrons	MANA	#	#	#	#	S16 18.715	E49 47.338	
435	Ealb	albifrons	MANA	#	#	#	#	S16 18.715	E49 47.338	
438	Ealb	albifrons	MANA	#	#	#	#	S16 18.715	E49 47.338	
487	Ealb	albifrons	ANJO	NA	NA	#	#	S14 11.095	E49 56.000	removed
491	Ealb	albifrons	ANJO	#	#	#	#	S14 11.095	E49 56.000	Phenotype inferred from CytB and locality
519	Ealb	albifrons	BEAL	#	#	#	#			pet
567	Ealb	albifrons	MARO	#	#	#	#	S14 11.095	E49 56.000	
570	Ealb	albifrons	MARO	#	#	#	#	S14 26.252	E49 46.612	
576	Ealb	albifrons	MARO	#	#	#	#	S14 26.071	E49 45.641	
582	Ealb	albifrons	MARO	#	#	#	#	S14 26.099	E49 46.023	

583	Ealb	albifrons	MARO	#	#	NA	#	S14 26.099	E49 46.023	
586	Ealb	albifrons	MARO	#	#	#	#	S14 26.252	E49 46.612	
242	Ecin	hybrid	ANDR	#	#	#	#	S22 08.714	E46 56.926	
245	Ecin	hybrid	ANDR	#	#	NA	#	S22 08.714	E46 56.926	
250	Ecin	hybrid	ANDR	#	#	#	#	S22 09.854	E46 56.367	
251	Ecinxrufi	hybrid	ANDR	NA	NA	NA	#	S22 09.854	E46 56.367	
253	Ecin	hybrid	ANDR	NA	NA	NA	#	S22 09.854	E46 56.367	
271	Ecin	cinereiceps	MANO	#	NA	NA	#	S23 01.200	E47 41.569	
396	Ecol	collaris	ANDO	NA	NA	NA	#	S24 45.330	E46 51.418	
397	Ecol	collaris	ANDO	#	#	#	#	S24 45.330	E46 51.418	
398	Ecol	collaris	MAND	#	#	#	#	S24 56.717	E46 59.752	
399	Ecol	collaris	MAND	#	#	#	#	S24 56.717	E46 59.752	
402	Ecol	collaris	MAND	#	#	#	#	S24 56.717	E46 59.752	
403	Ecol	collaris	MAND	#	#	#	#	S24 56.717	E46 59.752	
408	Ecol	collaris	MAND	#	#	#	#	S24 56.717	E46 59.752	
411	Ecol	collaris	STLU	#	#	#	#	S24 46.484	E47 10.354	
413	Ecol	collaris	STLU	#	#	#	#	S24 46.484	E47 10.354	
417	Ecol	collaris	STLU	#	#	#	#	S24 46.484	E47 10.354	
422	Ecol	collaris	STLU	NA	#	NA	#	S24 46.484	E47 10.354	
46	Ecor	coronatus	ANAL	#	#	#	#	S12 44.399	E49 29.076	
47	Ecor	coronatus	ANAL	NA	NA	NA	#	S12 44.399	E49 29.076	

50	Ecor	coronatus	DARA	NA	NA	NA	#	S13 10.026	E49 42.408	
78	Ecor	coronatus	ANKA	#	#	#	#	S12 56.389	E49 07.242	
490	Ecor	coronatus	ANJO	#	#	#	#	S14 11.095	E49 56.000	Phenotype inferred from CytB + vocalization
492	Ecor	coronatus	ANJO	#	#	#	#	S14 11.095	E49 56.000	Phenotype inferred from CytB + vocalization
520	Efla	flavifrons	MAVO	NA	NA	#	NA	S14 01.776	E48 16.149	removed
563	Efla	flavifrons	MAVO	NA	NA	NA	#	S14 01.661	E48 16.603	Phenotype inferred from CytB
565	Efla	flavifrons	MAVO	#	#	NA	#	S14 01.661	E48 16.603	unknown Phenotype
566	Efla	flavifrons	MAVO	NA	NA	NA	#	S14 01.661	E48 16.603	Phenotype inferred from CytB
92	Eful	fulvus	AMPI	#	#	#	#	S16 19.163	E46 48.374	
100	Eful	fulvus	AMPI	NA	NA	NA	#	S16 19.163	E46 48.374	
123	Eful	fulvus	AMPI	NA	NA	NA	#	S16 19.163	E46 48.374	
131	Eful	fulvus	AMPI	NA	NA	NA	#	S16 19.163	E46 48.374	
157	Eful	fulvus	TSIN	#	#	#	#	S19 43.121	E47 49.163	
159	Eful	fulvus	TSIN	NA	NA	NA	#	S19 43.121	E47 49.163	
161	Eful	fulvus	TSIN	#	#	#	#	S19 43.121	E47 49.163	
162	Eful	fulvus	TSIN	NA	NA	#	NA	S19 43.203	E47 49.078	
163	Eful	fulvus	TSIN	#	#	NA	#	S19 43.203	E47 49.078	
333	Eful	fulvus	ANDA	#	#	#	#	S18 56.298	E48 25.153	

334	Eful	fulvus	ANDA	#	#	NA	#	S18 56.298	E48 25.153	
335	Eful	fulvus	ANDA	#	#	#	#	S18 56.298	E48 25.153	
336	Eful	fulvus	ANDA	#	#	#	#	S18 56.298	E48 25.153	
345	Eful	fulvus	TAMP	NA	NA	NA	#	S17 17.163	E49 24.522	
350	Eful	fulvus	ANDA	NA	NA	NA	#	S18 56.298	E48 25.153	
352	Eful	fulvus	ANDA	NA	NA	NA	#	S18 56.298	E48 25.153	
353	Eful	fulvus	ANDA	#	#	#	#	S18 56.298	E48 25.153	
356	Eful	fulvus	ANDA	#	#	#	#	S18 56.298	E48 25.153	
474	Eful	fulvus	AMBO	#	#	#	#	S18 11.906	E47 16.929	
496	Eful	fulvus	MANG	#	#	#	#	S14 11.955	E48 57.093	
542	Eful	fulvus	ZAHA	#	#	#	#	S17 29.167	E48 44.007	
544	Eful	fulvus	ZAHA	NA	NA	NA	#	S17 28.987	E48 44.120	
558	Eful	fulvus	ZAHA	NA	NA	NA	#	S17 29.167	E48 44.007	
528	Emac	macaco	AMTO	#	#	#	NA	13 27.565	48 29.722	
529	Emac	macaco	AMTO	#	#	#	#	13 27.565	48 29.722	
14	Emon	mongoz	KATS	#	#	#	#	S15 42.917	E46 10.238	
27	Emon	mongoz	KATS	#	#	#	NA	S15 42.917	E46 10.238	
136	Emon	mongoz	MADI	NA	NA	NA	#	S16 22.626	E46 26.219	
375	Erub	rubriventer	RANO	#	#	#	#	S21 15.838	E47 25.329	
376	Erub	rubriventer	RANO	#	#	#	#	S21 15.838	E47 25.329	
380	Erub	rubriventer	FENA	#	#	#	#	S17 22.558	E49 24.319	

511	Erub	rubriventer	MANG	#	#	#	#	S14 11.955	E48 57.093	
164	Erufi	rufifrons	AMBA	#	#	#	#	S19 47.569	E44 39.331	
170	Erufi	rufifrons	AMBA	#	#	#	#	S19 54.574	E44 38.410	
172	Erufi	rufifrons	AMBA	#	#	#	#	S19 54.574	E44 38.410	
176	Erufi	rufifrons	KIRI	#	#	#	#	S20 04.290	E44 41.058	
181	Erufi	rufifrons	KIRI	#	#	NA	#	S20 04.290	E44 41.058	
184	Erufi	rufifrons	KIRI	#	#	#	NA	S20 04.290	E44 41.058	
189	Erufi	rufifrons	KIRI	#	#	#	#	S20 04.290	E44 41.058	
195	Erufi	rufifrons	KIRI	#	NA	#	#	S20 04.290	E44 41.058	
197	Erufi	rufifrons	KIRI	#	#	NA	#	S20 04.290	E44 41.058	
199	Erufi	rufifrons	KIRI	NA	NA	NA	#	S20 04.290	E44 41.058	
202	Erufi	rufifrons	KIRI	NA	NA	NA	#	S20 04.290	E44 41.058	
216	Erufi	rufifrons	KIRI	NA	NA	NA	#	S20 04.290	E44 41.058	
314	Erufi	rufifrons	BERE	#	#	#	#	S24 59.932	E46 17.874	
322	Erufi	rufifrons	BERE	#	#	#	#	S24 59.932	E46 17.874	
328	Erufi	rufifrons	MAND	#	#	NA	NA	S24 56.717	E46 59.752	
358	Erufi	rufifrons	RANO	#	#	#	#	S21 15.762	E47 25.364	
359	Erufi	rufifrons	RANO	#	#	#	#	S21 15.781	E47 25.429	
360	Erufi	rufifrons	RANO	#	#	#	#	S21 15.781	E47 25.429	
361	Erufi	rufifrons	RANO	#	#	#	#	S21 15.762	E47 25.364	
366	Erufi	rufifrons	RANO	#	#	#	#	S21 15.838	E47 25.329	

373	Erufi	rufifrons	RANO	#	#	#	#	S21 15.838	E47 25.329	
374	Erufi	rufifrons	RANO	NA	NA	NA	#	S21 15.838	E47 25.329	
448	Erufi	rufifrons	MAKA	#	#	#	#	S21 34.036	E45 04.228	
4	Erufu	rufus	KATS	NA	NA	NA	#	S15 42.955	E46 10.240	
16	Erufu	rufus	KATS	NA	NA	NA	#	S15 42.955	E46 10.240	
137	Erufu	rufus	MADI	#	#	#	#	S16 22.409	E46 26.118	
138	Erufu	rufus	MADI	#	#	#	#	S16 22.409	E46 26.118	
139	Erufu	rufus	MADI	#	#	#	#	S16 22.409	E46 26.118	
141	Erufu	rufus	MADI	#	#	#	#	S16 22.519	E46 26.285	
142	Erufu	rufus	MADI	#	#	#	#	S16 22.519	E46 26.285	
145	Erufu	rufus	MADI	#	#	#	#	S16 22.766	E46 26.384	
147	Erufu	rufus	MADI	NA	NA	NA	#	S16 22.935	E46 25.799	
425	Erufu	rufus	BEMA	NA	NA	NA	#	S19 01.134	E44 46.444	
431	Erufu	rufus	BEMA	#	NA	NA	NA	S19 01.134	E44 46.444	removed
432	Erufu	rufus	BEMA	#	NA	NA	NA	S19 01.134	E44 46.444	removed
440	Erufu	rufus	BEMA	#	#	#	#	S19 01.134	E44 46.444	
34	Esan	sanfordi	ANAL	#	#	NA	#	S12 44.241	E49 28.859	
35	Esan	sanfordi	ANAL	#	#	#	#	S12 44.241	E49 28.859	
54	Esan	sanfordi	MTDA	NA	NA	NA	#	S12 31.346	E49 10.398	
64	Esan	sanfordi	MTDA	#	#	#	#	S12 31.346	E49 10.398	
65	Esan	sanfordi	MTDA	#	#	#	#	S12 31.346	E49 10.398	

66	Esan	sanfordi	MTDA	#	#	#	#	S12 31.346	E49 10.398	
67	Esan	sanfordi	MTDA	#	#	#	#	S12 31.381	E49 10.395	
69	Esan	sanfordi	MTDA	NA	NA	NA	#	S12 31.346	E49 10.398	
72	Esan	sanfordi	MTDA	NA	NA	NA	#	S12 31.175	E49 10.488	
80	Esan	sanfordi	ANKA	NA	NA	NA	#	S12 56.486	E49 07.268	
81	Esan	sanfordi	ANKA	NA	NA	NA	#	S12 56.486	E49 07.268	
532	Esan	sanfordi	MAHA	#	#	#	#	S12 46.019	E48 59.966	
539	Esan	sanfordi	MAHA	#	#	NA	#	S12 46.019	E48 59.966	
NHM(ZD)1935 1887	Ealb	albifrons	NA	NA	NA	NA	#	Maroantsetra		
NHMB44661	Ealb	albifrons	NA	NA	NA	NA	#	Maroantsetra		
MCZ44886	Ecol	collaris	NA	NA	NA	NA	#	Ivondro		
MCZ44887	Ecol	collaris	NA	NA	NA	NA	#	Manangotry		
MCZ44890	Ecol	collaris	NA	NA	NA	NA	#	Manangotry		
MCZ44892	Ecol	collaris	NA	NA	NA	NA	#	Fanjahira		
MCZ44895	Ecol	collaris	NA	NA	NA	NA	#	Fort Dauphin		
MCZ44896	Ecol	collaris	NA	NA	NA	NA	#	Fort Dauphin		
NHMB44671	Eful	fulvus	NA	NA	NA	NA	#	Andapa		
MCZ16371	Eful	fulvus	NA	NA	NA	NA	#	Didy		
MCZ8044	Eful	fulvus	NA	NA	NA	NA	#	northwest Madagascar		
NHMB44478	Eful	fulvus	NA	NA	NA	NA	#	Anaborano		
NHMB83848	Eful	fulvus	NA	NA	NA	NA	#	Sakana		

NHM(ZD)1935 1876	Eful	fulvus	NA	NA	NA	NA	#	Lokosy	
NHM(ZD)1948 148	Eful	fulvus	NA	NA	NA	NA	#	Imerimandroso	
MCZ44901	Emac	macaco	NA	NA	NA	NA	#	Valley of Ramena	
NHMB7935	Emac	macaco	NA	NA	NA	NA	#	NA	
NHMB83839	Emac	macaco	NA	NA	NA	NA	#	Nosy Be	
NHMB83840	Emac	macaco	NA	NA	NA	NA	#	Nosy Be	
NHMB83841	Emac	macaco	NA	NA	NA	NA	#	Lokobe	
NHMB83842	Emac	macaco	NA	NA	NA	NA	#	Vavatobe	
MCZ44894	Emon	mongoz	NA	NA	NA	NA	#	80 km south of Mahajnaga	
NHMB44477	Emon	mongoz	NA	NA	NA	NA	#	Lokobe	
MCZ44898	Erub	rubriventer	NA	NA	NA	NA	#	Antsianaka	
NHM19351811 5	Erufi	rufifrons	NA	NA	NA	NA	#	Lokosy	
MCZ16354	Erufi	rufifrons	NA	NA	NA	NA	#	Upper Tsiribihina	
MCZ16356	Erufi	rufifrons	NA	NA	NA	NA	#	Upper Tsiribihina	
MCZ16357	Erufi	rufifrons	NA	NA	NA	NA	#	Upper Tsiribihina	
NHM(ZD)1882 314	Erufi	rufifrons	NA	NA	NA	NA	#	Fianarantsoa	
NHMB89006	Erufi	rufifrons	NA	NA	NA	NA	#	Tabiky	
MCZ44897	Erufu	rufus	NA	NA	NA	NA	#	80 km south of Mahajanga	

NHM(ZD)1870 5527	Esan	sanfordi	NA	NA	NA	NA	#	Vohemar	
	Lcat	Lemur catta					JF489136		

Table S2: Museum specimen used for morphometric analysis. AMNH= American Museum of National History, New York; USNM= Smithsonian Institution Washington D.C.; NHM= National History Museum, London; MCZ= Museum of Comparative Zoology, Boston. m= male, f= female

ID	Species	Sex	Locality	Museum
AMNH100566	albifrons	f	Maroantsetra	AMNH
AMNH100572	albifrons	f	Maroantsetra	AMNH
AMNH100586	albifrons	m	Maroantsetra	AMNH
AMNH100587	albifrons	m	Andapa	AMNH
AMNH100588	albifrons	m	Maroantsetra	AMNH
AMNH100589	albifrons	m	Maroantsetra	AMNH
AMNH170699	albifrons	m	Ambatondrama	AMNH
AMNH170701	albifrons	f	Ambatondrama	AMNH
AMNH170708	albifrons	f	Ambatondrama	AMNH
AMNH170715	albifrons	f	Ambatondrama	AMNH
AMNH170717	albifrons	f	Ambatondrama	AMNH
AMNH170719	albifrons	f	Ambatondrama	AMNH
AMNH170723	albifrons	f	Ambatondrama	AMNH
AMNH170725	albifrons	f	Ambatondrama	AMNH
AMNH170728	albifrons	f	Ambatondrama	AMNH
AMNH170731	albifrons	m	Ambatondrama	AMNH
ZD19351887	albifrons	m	Maroantsetra	NHM

ZD19351888	albifrons	m	Maroantsetra	NHM
ZD19351890	albifrons	m	Maroantsetra	NHM
ZD19351892	albifrons	m	Maroantsetra	NHM
ZD19351893	albifrons	f	Maroantsetra	NHM
ZD19351894	albifrons	f	Maroantsetra	NHM
ZD19351895	albifrons	f	Maroantsetra	NHM
ZD19351896	albifrons	f	Maroantsetra	NHM
ZD19351897	albifrons	f	Maroantsetra	NHM
ZD19351898	albifrons	f	Maroantsetra	NHM
ZD19351899	albifrons	m	Andapa	NHM
AMNH100561	cinereiceps	f	Manombo	AMNH
AMNH100562	cinereiceps	m	Vondrozo	AMNH
ZD193518112	cinereiceps	m	Manombo	NHM
AMNH100818	cinereiceps	f	Vondrozo	AMNH
AMNH170749	collaris	f	Eminiminy	AMNH
AMNH170750	collaris	f	Eminiminy	AMNH
AMNH170755	collaris	f	Eminiminy	AMNH
AMNH170759	collaris	m	Eminiminy	AMNH
AMNH170764	collaris	f	Eminiminy	AMNH
AMNH170766	collaris	m	Eminiminy	AMNH

AMNH170770	collaris	f	Eminiminy	AMNH
AMNH170771	collaris	f	Eminiminy	AMNH
AMNH170772	collaris	m	Eminiminy	AMNH
MCZ44887	collaris		Manongotry	MCZ
MCZ44888	collaris		Manongotry	MCZ
MCZ44889	collaris		Manongotry	MCZ
AMNH100520	coronatus	f	Vohemar	AMNH
AMNH100610	coronatus	m	Vohemar	AMNH
AMNH100611	coronatus	m	Vohemar	AMNH
ZD19351859	coronatus	f	Vohemar	NHM
USNM63339	fulvus	m	Ambatobato near Tamatave	USNM
USNM63340	fulvus	m	Ambatobato near Tamatave	USNM
USNM63341	fulvus	f	Ambatobato near Tamatave	USNM
ZD1925833	fulvus	m	Lakato	NHM
ZD1925835	fulvus	m	Lakato	NHM
ZD19351850	mongoz	m	Ambararatabe	NHM
ZD19351852	mongoz	f	Ambararatabe	NHM
MCZ44881	rubriventer	m	Manonga	MCZ
MCZ44898	rubriventer	f	Antsianaka	NHM

MCZ44899	rubriventer	m	Antsianaka	NHM
MCZ8045	rubriventer			NHM
USNM63335	rubriventer	f		USNM
ZD18887241	rubriventer		Tamatave	NHM
ZD18979110	rubriventer	m	Vinanitelo	NHM
ZD1897919	rubriventer		Tanala	NHM
ZD19351846	rubriventer	f	Andapa	NHM
ZD19351847	rubriventer	f	Andapa	NHM
MCZ16356	rufifrons	f	30 miles south of Berevo	MCZ
MCZ16394	rufifrons	m	30 miles south of Berevo	MCZ
ZD18314	rufifrons		Fianarantsoa	NHM
ZD18314	rufifrons		Fianarantsoa	NHM
ZD19351876	rufifrons		Lokosy	NHM
ZD19391268	rufifrons	m	Manakara	NHM
ZD19391269	rufifrons	f	Manakara	NHM
ZD1948149	rufifrons	f	Beroboka	NHM
AMNH100524	rufifrons	m	Tabiky	AMNH
MCZ16353	rufifrons	m	Upper Siribihina, Bemara Gorges	MCZ

MCZ16354	rufifrons	m	Upper Siribihina Bemara Gorges	MCZ
MCZ16357	rufifrons	f	Upper Siribihina	MCZ
MCZ16365	rufifrons	m	Upper Siribihina	MCZ
MCZ16370	rufifrons	f	Upper Siribihina	MCZ
MCZ16393	rufifrons	f	Upper Siribihina	MCZ
MCZ16395	rufifrons	m	Upper Siribihina	MCZ
ZD1913341	rufus	f	Ambohimanga	NHM
ZD19351881	rufus	f	Tsiandro	NHM
ZD19351883	rufus	m	Namoroka	NHM
AMNH100532	rufus	f	Bekipany	AMNH
AMNH100607	rufus	m	near Ankoja	AMNH
AMNH100614	rufus	f	Ankoja	AMNH
AMNH100819	rufus	f	Namoroka	AMNH
AMNH100521	sanfordi	m	Tsarakibany	AMNH
ZD19351869	sanfordi	m	MtDambre	NHM
ZD19351871	sanfordi	m	MtDambre	NHM
ZD19351872	sanfordi	f	MtDambre	NHM
AMNH100518	sanfordi	f	MtDambre	AMNH
AMNH100577	sanfordi	f	MtDambre	AMNH

Table S3: Museum specimen used for pelage color analysis. AMNH= American Museum of National History, New York; USNM= Smithsonian Institution Washington D.C.; NHM= National History Museum, London; MCZ= Museum of Comparative Zoology, Boston. m= male, f= female

ID	Species	Locality	Sex	Museum
AMNH100486	albifrons	Maroantsetra	m	AMNH
AMNH100558	albifrons	Maroantsetra	m	AMNH
AMNH100587	albifrons	Andapa	m	AMNH
AMNH100588	albifrons	Maroantsetra	m	AMNH
AMNH100589	albifrons	Maroantsetra	m	AMNH
AMNH100590	albifrons	Maroantsetra	m	AMNH
USNM63344	albifrons	RiverFaraony	m	USNM
ZD193518103	cinereiceps	Vondrozo	m	NHM
AMNH100562	cinereiceps	Manombo	m	AMNH
AMNH100573	cinereiceps	Vondrozo	m	AMNH
AMNH100579	cinereiceps	Vondrozo	m	AMNH
AMNH100602	cinereiceps	Vondrozo	m	AMNH
ZD193518100	cinereiceps	Vondrozo	m	NHM
ZD193518101	cinereiceps	Vondrozo	m	NHM
ZD193518102	cinereiceps	Vondrozo	m	NHM
ZD193518104	cinereiceps	Vondrozo	m	NHM
ZD193518105	cinereiceps	Vondrozo	m	NHM
AMNH170751	collaris	Eminiminy	m	AMNH

AMNH170760	collaris	Eminiminy	m	AMNH
AMNH170764	collaris	Eminiminy	m	AMNH
AMNH170765	collaris	Eminiminy	m	AMNH
MCZ44893	collaris	Fanjahira	m	MCZ
MCZ44895	collaris	Fanjahira	m	MCZ
USNM317960	collaris	Bemangidy	m	USNM
USNM317961	collaris	Bemangidy	m	USNM
AMNH100527	fulvus	Ivohibe	m	AMNH
AMNH100528	fulvus	Ivohibe	m	AMNH
USNM63339	fulvus	Ambatobato	m	AMNH
USNM63340	fulvus	Ambatobato	m	AMNH
ZD18823123	rufifrons	Fianarantsoa	m	NHM
ZD19351879	rufifrons	Tabiky	m	NHM
ZD19351882	rufus	Ankoja	m	NHM
ZD19351886	rufifrons	Ivohibe	m	NHM
AMNH100519	rufifrons	Tabiky	m	AMNH
AMNH100524	rufifrons	Tabiky	m	AMNH
MCZ16394	rufifrons	30msouthofBerevo	m	MCZ
MCZ16365	rufifrons	UpperSiribihinariver	m	MCZ
MCZ16395	rufifrons	UpperSiribihinariver	m	MCZ

MCZ16354	rufifrons	UpperSiribihinariver	m	MCZ
MCZ16353	rufifrons	UpperSiribihinariver	m	MCZ
MCZ16355	rufifrons	UpperSiribihinariver	m	MCZ
USNM63338	rufifrons	Fianarantsoa	m	AMNH
AMNH100517	rufus	Soalala	m	AMNH
AMNH100522	rufus	Namoroka	m	AMNH
AMNH100523	rufus	Namoroka	m	AMNH
AMNH100525	rufus	Soalala	m	AMNH
AMNH100569	rufus	Tsitampiky	m	AMNH
AMNH100607	rufus	Ankoja	m	AMNH
AMNH100521	sanfordi	Tsarakibany	m	AMNH
AMNH100585	sanfordi	MtDambre	m	AMNH
ZD18705527	sanfordi	Vohemar	m	NHM
ZD19351869	sanfordi	MtDambre	m	NHM
ZD19351870	sanfordi	MtDambre	m	NHM
ZD19351871	sanfordi	MtDambre	m	NHM
AMMH170725	albifrons	Ambatondrandama	f	AMNH
AMNH100559	albifrons		f	AMNH
AMNH100560	albifrons	Maroantsetra	f	AMNH
AMNH100566	albifrons		f	AMNH

AMNH100572	albifrons	Maroantsetra	f	AMNH
AMNH170705	albifrons	Ambatondrandama	f	AMNH
AMNH170708	albifrons	Ambatondrandama	f	AMNH
AMNH170715	albifrons	Ambatondrandama	f	AMNH
AMNH170717	albifrons	Ambatondrandama	f	AMNH
AMNH170720	albifrons	Ambatondrandama	f	AMNH
AMNH170723	albifrons	Ambatondrandama	f	AMNH
AMNH170728	albifrons	Ambatondrandama	f	AMNH
AMNH100561	cinereiceps		f	AMNH
AMNH100564	cinereiceps		f	AMNH
AMNH100565	cinereiceps		f	AMNH
AMNH100568	cinereiceps		f	AMNH
AMNH100570	cinereiceps		f	AMNH
AMNH100575	cinereiceps		f	AMNH
AMNH100576	cinereiceps	Vondrozo	f	AMNH
AMNH100580	cinereiceps		f	AMNH
AMNH100581	cinereiceps		f	AMNH
ZD193518106	cinereiceps		f	NHM
ZD193518107	cinereiceps		f	NHM
ZD193518108	cinereiceps	Vondrozo	f	London

ZD193518109	cinereiceps		f	NHM
ZD193518110	cinereiceps		f	NHM
ZD193518111	cinereiceps		f	NHM
ZD193518113	cinereiceps		f	NHM
AMNH170750	collaris		f	AMNH
AMNH170755	collaris		f	AMNH
AMNH170771	collaris		f	AMNH
AMNH100529	fulvus		f	AMNH
MCZ16371	fulvus		f	AMNH
USNM63341	fulvus		f	AMNH
USNM63342	fulvus		f	AMNH
USNM63343	fulvus		f	AMNH
ZD1913341	fulvus	Ambohimanga	f	NHM
AMNH100526	rufifrons	Ivohibe	f	AMNH
AMNH100571	rufifrons	Tabiky/Ankazoabo	f	NHM
AMNH100582	rufifrons	Tabiky/Ankazoabo	f	AMNH
MCZ16356	rufifrons	30m south of Berevo	f	MCZ
ZD1882311	rufifrons	Fianarantsoa	f	NHM
ZD19351877	rufifrons	Tabiky/Ankazoabo	f	NHM
ZD19351878	rufifrons	Tabiky/Ankazoabo	f	NHM

ZD19391269	rufifrons	Manakara	f	NHM
ZD1948149	rufifrons	Beroboka	f	NHM
AMNH100532	rufus	Bekipany	f	AMNH
AMNH100614	rufus	Ankoja	f	AMNH
MCZ18630	rufus	Upper Siribihina river	f	MCZ
MCZ18630	rufus	80km south of Majunga	f	MCZ
ZD18911225	rufus	near Majunga	f	NHM
ZD19351881	rufus	Tsiandro	f	NHM
AMNH100518	sanfordi	MtDambre	f	AMNH
AMNH100577	sanfordi	MtDambre	f	AMNH
AMNH100578	sanfordi	MtDambre	f	AMNH
ZD19351872	sanfordi	MtDambre	f	NHM
ZD19351873	sanfordi	MtDambre	f	NHM

Table S4: Primer and annealing temperatures used in this study. MID= Multiplexidentfier, °C= Annealing temperature.

Primer	454- Adaptor	MID	Universal tail	Template specific	Comment	°C
vWF forward			GTAAAACGACGGC CAGT	CTTCTCCATTGTC ATTGAGAC	Template specific primer with universal tale for 1st PCR	60
vWF reverse			AACAGCTATGACC ATG	AGCTTCRCACAGC AGAGACT	Template specific primer with universal tale for 1st PCR	60
NRAMP forward			GTAAAACGACGGC CAGT	TAATCCTGCTGTCT CCTGAC	Template specific primer with universal tale for 1st PCR	60
NRAMP revers			AACAGCTATGACC ATG	GGGATTCTGAAAC CAGAGTG	Template specific primer with universal tale for 1st PCR	60
ENO forward			GTAAAACGACGGC CAGT	GCGCCACACTAAA TGACTTG	Template specific primer with universal tale for 1st PCR	60
ENO reverse			AACAGCTATGACC ATG	CTGCCTCATGCCA CTCTCA	Template specific primer with universal tale for 1st PCR	60
For MID 1	CGTATCGCCTCCC TCGCGCCATCAG	ACGAGTGCGT	GTAAAACGACGGC CAGT		454 Primer for 2nd PCR	60
For MID 2	CGTATCGCCTCCC TCGCGCCATCAG	ACGCTCGACA	GTAAAACGACGGC CAGT		454 Primer for 2nd PCR	60
For MID 3	CGTATCGCCTCCC TCGCGCCATCAG	AGACGCACTC	GTAAAACGACGGC CAGT		454 Primer for 2nd PCR	60
For MID 4	CGTATCGCCTCCC TCGCGCCATCAG	AGCACTGTAG	GTAAAACGACGGC CAGT		454 Primer for 2nd PCR	60
For MID 5	CGTATCGCCTCCC TCGCGCCATCAG	ATCAGACACG	GTAAAACGACGGC CAGT		454 Primer for 2nd PCR	60
For MID 6	CGTATCGCCTCCC TCGCGCCATCAG	ATATCGCGAG	GTAAAACGACGGC CAGT		454 Primer for 2nd PCR	60

For MID 7	CGTATCGCCTCCC TCGCGCCATCAG	CGTGTCTCTA	GTAAAACGACGGC CAGT		454 Primer for 2nd PCR	60
For MID 8	CGTATCGCCTCCC TCGCGCCATCAG	CTCGCGTGTC	GTAAAACGACGGC CAGT		454 Primer for 2nd PCR	60
For MID 9	CGTATCGCCTCCC TCGCGCCATCAG	TAGTATCAGC	GTAAAACGACGGC CAGT		454 Primer for 2nd PCR	60
For MID 10	CGTATCGCCTCCC TCGCGCCATCAG	TCTCTATGCG	GTAAAACGACGGC CAGT		454 Primer for 2nd PCR	60
Rev MID 1	CTATGCGCCTTGC CAGCCCGCTCAG	ACGAGTGCGT	AACAGCTATGACC ATG		454 Primer for 2nd PCR	60
Rev MID 2	CTATGCGCCTTGC CAGCCCGCTCAG	ACGCTCGACA	AACAGCTATGACC ATG		454 Primer for 2nd PCR	60
Rev MID 3	CTATGCGCCTTGC CAGCCCGCTCAG	AGACGCACTC	AACAGCTATGACC ATG		454 Primer for 2nd PCR	60
Rev MID 4	CTATGCGCCTTGC CAGCCCGCTCAG	AGCACTGTAG	AACAGCTATGACC ATG		454 Primer for 2nd PCR	60
Rev MID 5	CTATGCGCCTTGC CAGCCCGCTCAG	ATCAGACACG	AACAGCTATGACC ATG		454 Primer for 2nd PCR	60
Rev MID 6	CTATGCGCCTTGC CAGCCCGCTCAG	ATATCGCGAG	AACAGCTATGACC ATG		454 Primer for 2nd PCR	60
Rev MID 7	CTATGCGCCTTGC CAGCCCGCTCAG	CGTGTCTCTA	AACAGCTATGACC ATG		454 Primer for 2nd PCR	60
Rev MID 8	CTATGCGCCTTGC CAGCCCGCTCAG	CTCGCGTGTC	AACAGCTATGACC ATG		454 Primer for 2nd PCR	60
Rev MID 9	CTATGCGCCTTGC CAGCCCGCTCAG	TAGTATCAGC	AACAGCTATGACC ATG		454 Primer for 2nd PCR	60

Rev MID 10	CTATGCGCCTTGC CAGCCCGCTCAG	TCTCTATGCG	AACAGCTATGACC ATG		454 Primer for 2nd PCR	60
CytB_fulvus_group_r ev1				CCTCATGGAAGGA CATATCC	CytB- museum samples Ealb, Ecol, Esan, Eful, Ecin	58
CytB_fulvus_group_r ev2				TCCTCATGGAAGG ACATACC	CytB- museum samples Erufi, Erufu, Eful	58
CytB_outgroup_for				AYAGCAGACACAA CAACCGC	CytB- museum samples Emac, Erub, Ecor, Emon	58
CytB_outgroup_rev				TCCTCATGGGAGR ACRTACC	CytB- museum samples Emac, Erub, Ecor, Emon	58
CytB_fulvus_group_f or				ACAGCAGACACAA CAACAGC	CytB- museum + fecal samples	60
CytB_for_94				AAYTTCGGTTCCC TCCTAGG	CytB- fecal samples	60
CytB_rev_173				TCTGCTGTGTAGT GTATTGC	CytB- fecal samples	60
CytB_rev_413				ATTTGTCCYCATG GAAGGAC	CytB- fecal samples	60
CytB_for_436				ATTACAAAYCTCCT CTCAGC	CytB- fecal samples	60
CytB_rev_507				GGAGAAACCDCCT CAGATTC	CytB- fecal samples	60
CytB_for_610				GGATCTAAYAACC CACTAGG	CytB- fecal samples	60
CytB_rev_642				GTCTGATGARGTT CCTAGTGG	CytB- fecal samples	60

CytB_for_757				CCCGACAACTACA CACCAGC	CytB- fecal samples	60
CytB_rev_895				GGATAGAGARGAT TAGGGC	CytB- fecal samples	60
CytB_for_937				CGAAGCATATTATT CCGACCCC	CytB- fecal samples	60
CytB_rev_1034				TATTCGACGGGTT GGCCTCC	CytB- fecal samples	60

Table S5: PCR reaction mixtures.

	Cytochrome B		454 (nuclear)	
	ancient	feces	1st PCR	2nd PCR
Ingredient	μl			
Water	7,3	13,9	13,9	22,9
Hifi Buffer/ MasterMixPlus	12,5	3,0	3,0	3,0
DMSO		0,6	0,6	0,6
MgCl2 (15mM)		1,3	1,3	1,3
dNTPs	0,6	0,6	0,6	0,6
Primer A	0,1	0,15	0,15	0,15
Primer B	0,1	0,15	0,15	0,15
Taq		0,3	0,3	0,3
Template	5,0	10,0	10,0	1,0
Total	25,0	30,0	30,0	30,0

Table S6: fdr- corrected p- values for pairwise comparisons after permutational MANOVA of loud calls. n.s.= not significant.

**chucks**	albifrons	cinereiceps	collaris	fulvus	rufifrons	rufus	sanfordi
albifrons							
cinereiceps	< 0,05						
collaris	< 0,01	< 0,05					
fulvus	n.s.	n.s.	< 0,001				
rufifrons	n.s.	< 0,01	< 0,001	< 0,001			
rufus	n.s.	n.s.	< 0,001	< 0,05	< 0,001		
sanfordi	n.s.	< 0,01	< 0,001	n.s.	< 0,001	< 0,01	

**croaks**	albifrons	cinereiceps	collaris	fulvus	rufifrons	rufus
albifrons						
cinereiceps	n.s.					
collaris	n.s.	n.s.				
fulvus	n.s.	n.s.	< 0,01			
rufifrons	n.s.	n.s.	< 0,01	n.s.		
rufus	n.s.	n.s.	n.s.	n.s.	n.s.	
sanfordi	n.s.	n.s.	n.s.	n.s.	n.s.	n.s.

Table S7: FDR- corrected p-values for pairwise comparison of shapes. n.s.= not significant.

	albifrons	cinereiceps	collaris	coronatus	fulvus	mongoz	rubriventer	rufifrons	rufus
albifrons									
cinereiceps	< 0,05								
collaris	< 0,01	n.s.							
coronatus	< 0,001	< 0,001	< 0,001						
fulvus	< 0,05	n.s.	< 0,05	< 0,001					
mongoz	< 0,001	< 0,001	< 0,001	< 0,001	< 0,001				
rubriventer	< 0,001	< 0,01	< 0,001	< 0,001	< 0,001	< 0,001			
rufifrons	< 0,001	n.s.	< 0,01	< 0,01	< 0,05	< 0,001	< 0,001		
rufus	< 0,05	n.s.	< 0,05	< 0,001	n.s.	< 0,001	< 0,001	n.s.	
sanfordi	0,068	n.s.	n.s.	< 0,001	0,050	< 0,001	< 0,01	< 0,05	n.s.

Table S8: FDR-corrected p-values for pairwise comparisons of permutational MANOVA for pelage coloration. n.s.= not significant.

**females**	albifrons	cinereiceps	collaris	fulvus	rufifrons	rufus	sanfordi
albifrons							
cinereiceps	n.s.						
collaris	n.s.	n.s.					
fulvus	< 0,05	< 0,01	n.s.				
rufifrons	< 0,001	< 0,001	< 0,05	< 0,01			
rufus	< 0,001	< 0,001	n.s.	< 0,05	n.s.		
sanfordi	< 0,01	< 0,001	n.s.	< 0,05	n.s.	n.s.	

**males**	albifrons	cinereiceps	collaris	fulvus	rufifrons	rufus	sanfordi
albifrons							
cinereiceps	< 0,001						
collaris	< 0,001	n.s.					
fulvus	< 0,01	n.s.	n.s.				
rufifrons	< 0,001	< 0,001	< 0,01	< 0,05			
rufus	< 0,01	< 0,001	< 0,001	< 0,01	< 0,05		
sanfordi	< 0,01	< 0,001	< 0,01	< 0,01	n.s.	< 0,05	
