# Supplementary material for: True lemurs…true species - species delimitation using multiple data sources in the brown lemur complex
Source: BMC Evol Biol. 2013 Oct 26;13:233. doi: 10.1186/1471-2148-13-233 (PMC3819746; doi:10.1186/1471-2148-13-233)
Supplement: Additional file 2 — Figure S1. Spectrogram of typical disturbance and advertisement call of members of the fulvus group. X-axes= Time in seconds, y-axes= Frequency in kHz. Figure S2. 17 homologous landmarks used for geometric morphometric analyses. 1= Prosthion, 2= Posteriormost point of the left incisive foramen, 3= Premaxilla- maxilla suture, 4= Meeting point of premaxilla- maxilla suture and canine, 5= Posteriormost point of canine alveolus, 6= Maxilla- palatine suture, 7=Staphilio, 8= Posterior-jugal contact of alveolar ridge and 1st molar , 9= Lateralmost point of orbitum, 10= Lateralmostpoint of jugale, 11= Medialmostpoint of the braincase, 12= Lateralmostpoint of basisphenoid- vomer suture, 13= Lateralmostpoint of basioccipitale- basisphenoid suture 14= Lateralmostpoint of the meatus acousticus externus, 15= Basion, 16= Lateralmostpoint of foramen magnum, 17= Inion. Figure S3. 50 x 50 pixels measured with rectangular marqee tool in Adobe Photoshop. Figure S4. Scatterplot of bgPCA of morphological shape analysis including E. coronatus, E. mongoz and E. rubriventer. Points represent individuals along the first and second principal component. A color legend for the different species is given inside the plot. p= < 0.001 (999 randomizations). Figure S5. Neighbor joiningtree of the cytb locus including museum samples. Figure S6a-c. Bayesian gene trees of nuclear loci. a) eno, b) nramp c) vwf. [file 1471-2148-13-233-S2.pdf]

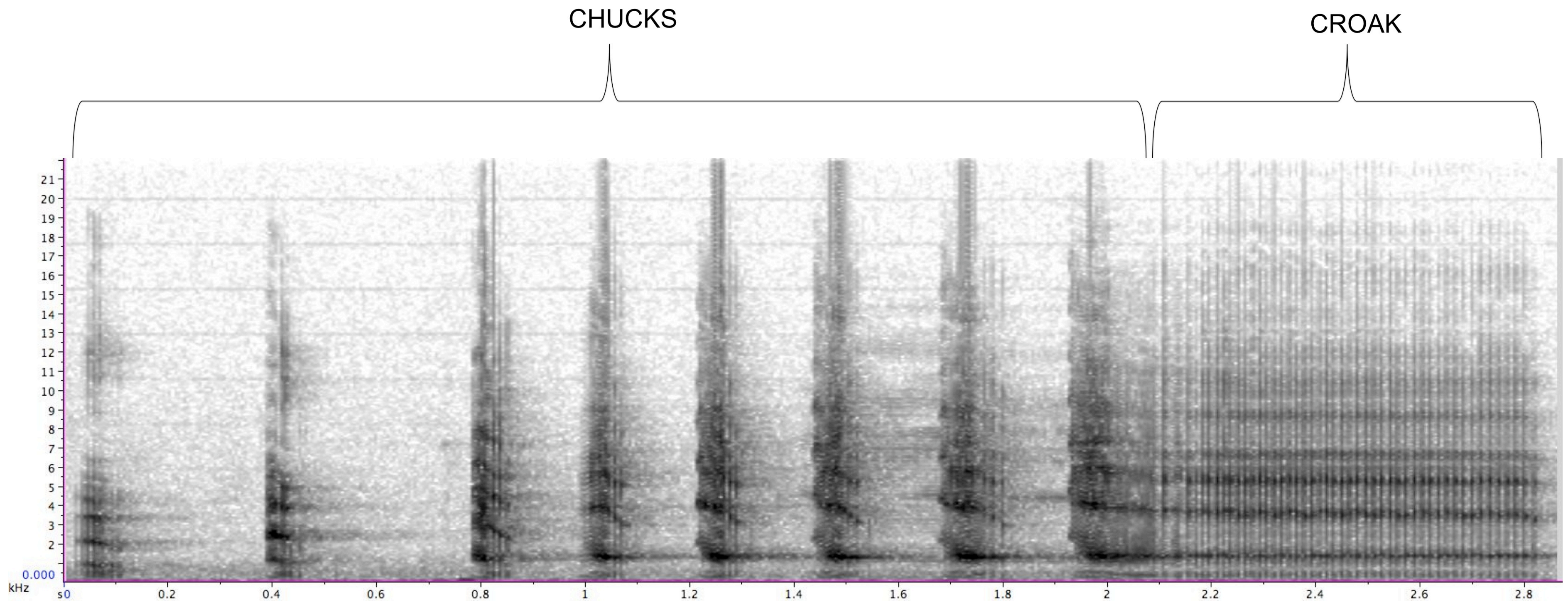

Figure S1: Spectrogram of typical disturbance and advertisement call of members of the fulvus group. X-axes= Time in seconds, y-axes= Frequency in kHz.

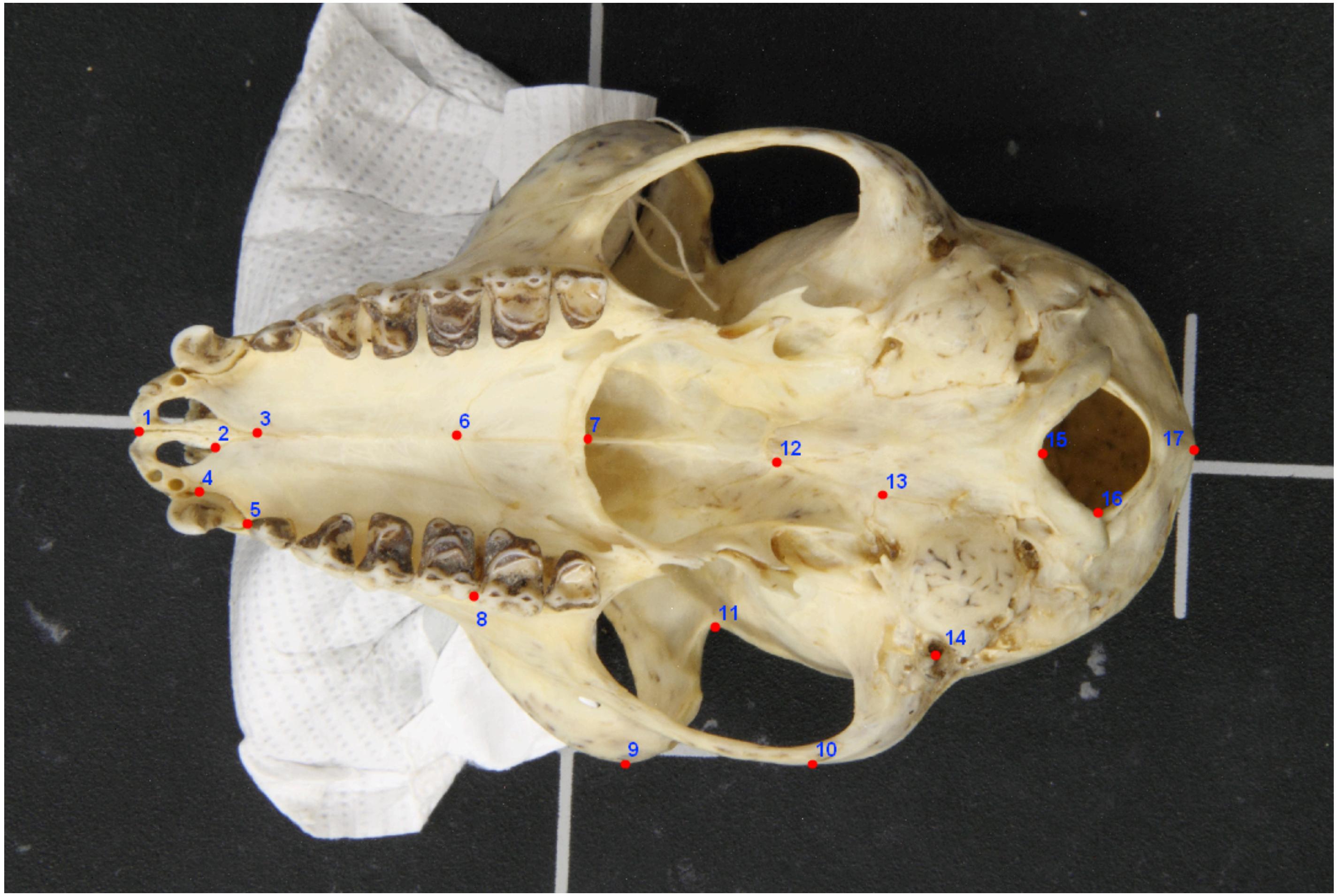

Figure S2: 17 homologous landmarks used for geometric morphometric analyses. 1= Prosthion, 2= Posteriormost point of the left incisive foramen, 3= Premaxilla- maxilla suture, 4= Meeting point of premaxilla- maxilla suture and canine, 5= Posteriormost point of canine alveolus, 6= Maxilla- palatine suture, 7=Staphilio, 8= Posterior-jugal contact of alveolar ridge and 1st molar , 9= Lateralmost point of orbitum, 10= Lateralmostpoint of jugale, 11= Medialmostpoint of the braincase, 12= Lateralmostpoint of basisphenoid- vomer suture, 13= Lateralmostpoint of basioccipitale- basisphenoid suture 14= Lateralmostpoint of the meatus acousticus externus, 15= Basion, 16= Lateralmostpoint of foramen magnum, 17= Inion

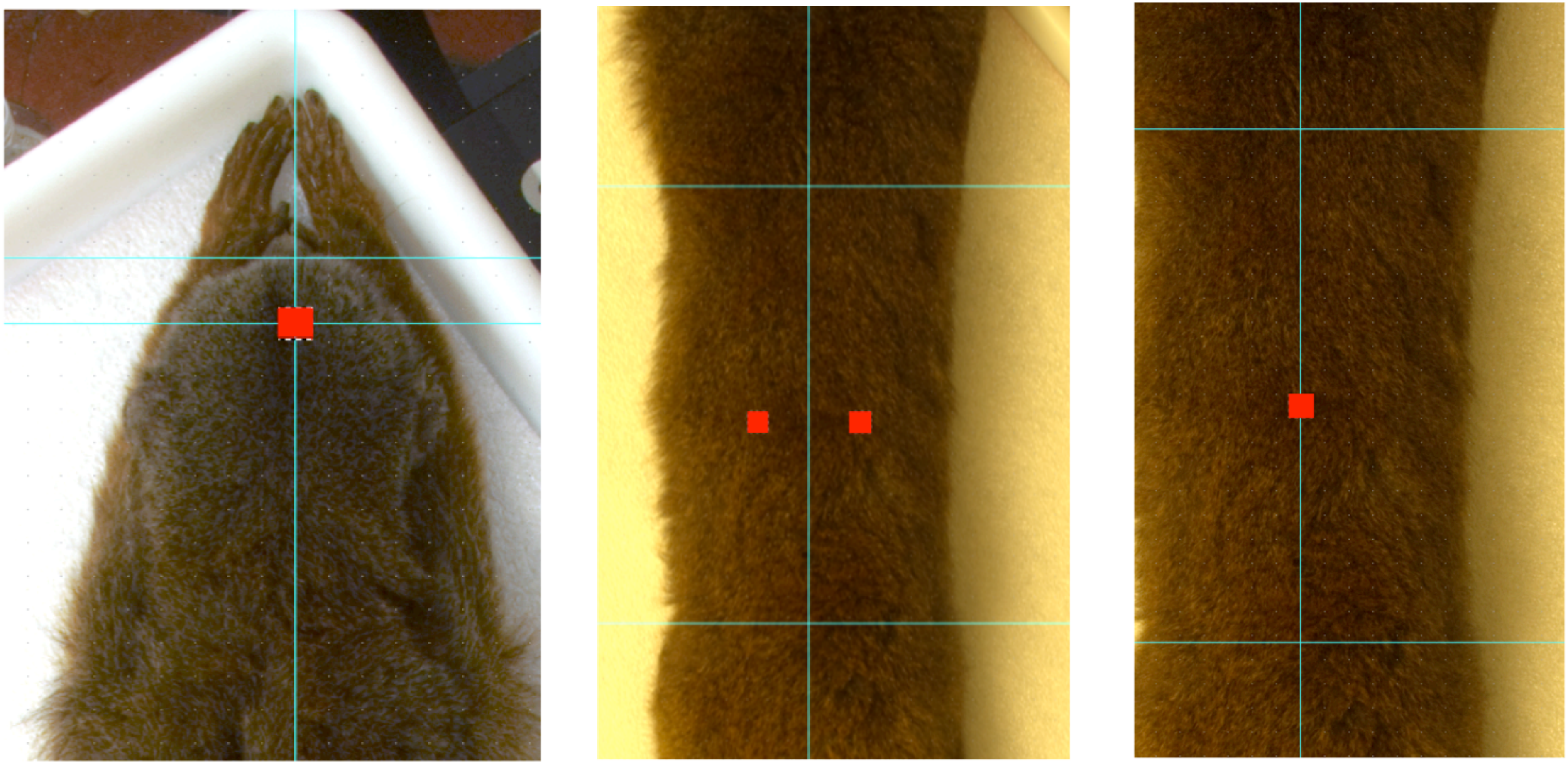

Figure S3: 50 x 50 pixels measured with rectangular marqee tool in Adobe Photoshop.

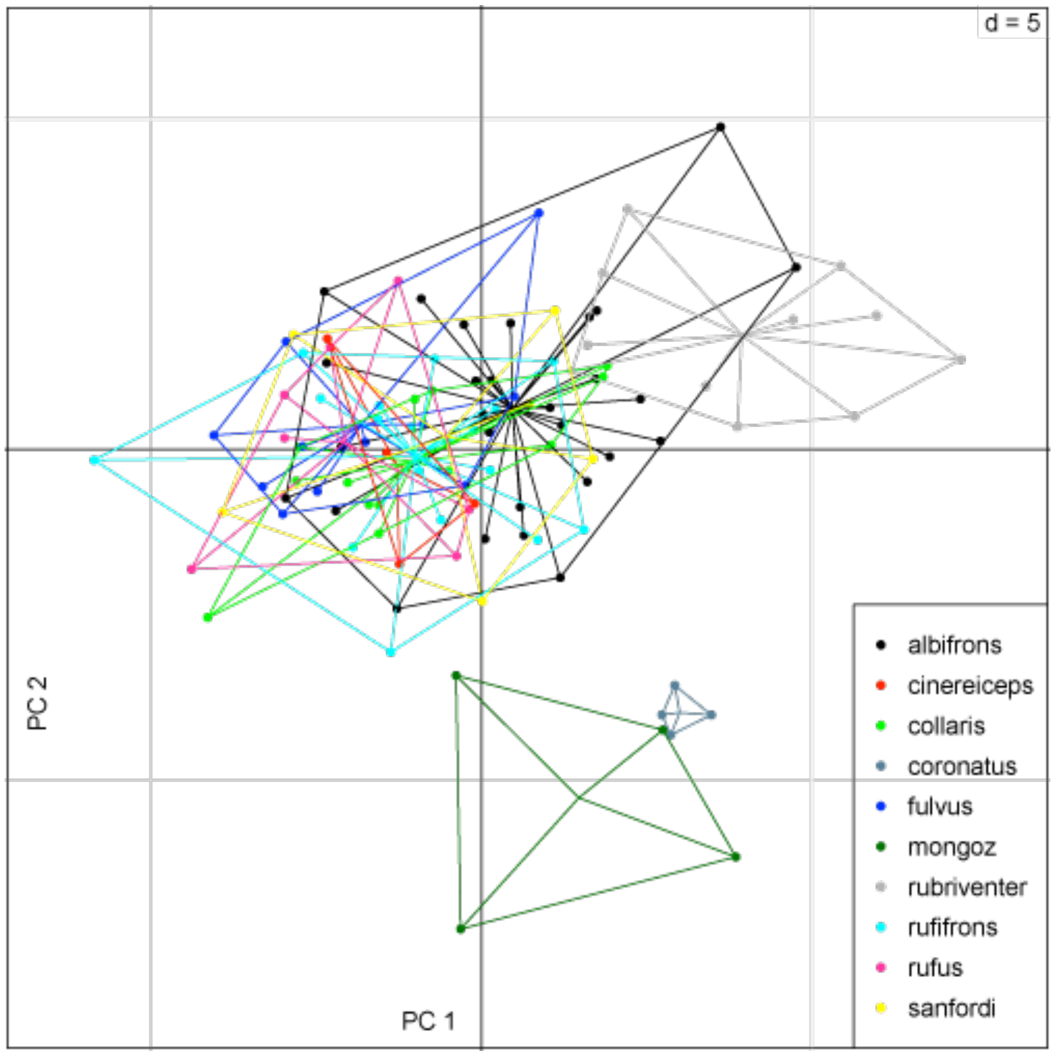

Figure S4: Scatterplot of bgPCA of morphological shape analysis including *E. coronatus, E. mongoz* and *E. rubriventer.* Points represent individuals along the first and second principal component. A color legend for the different species is given inside the plot. p= < 0.001 (999 randomizations)

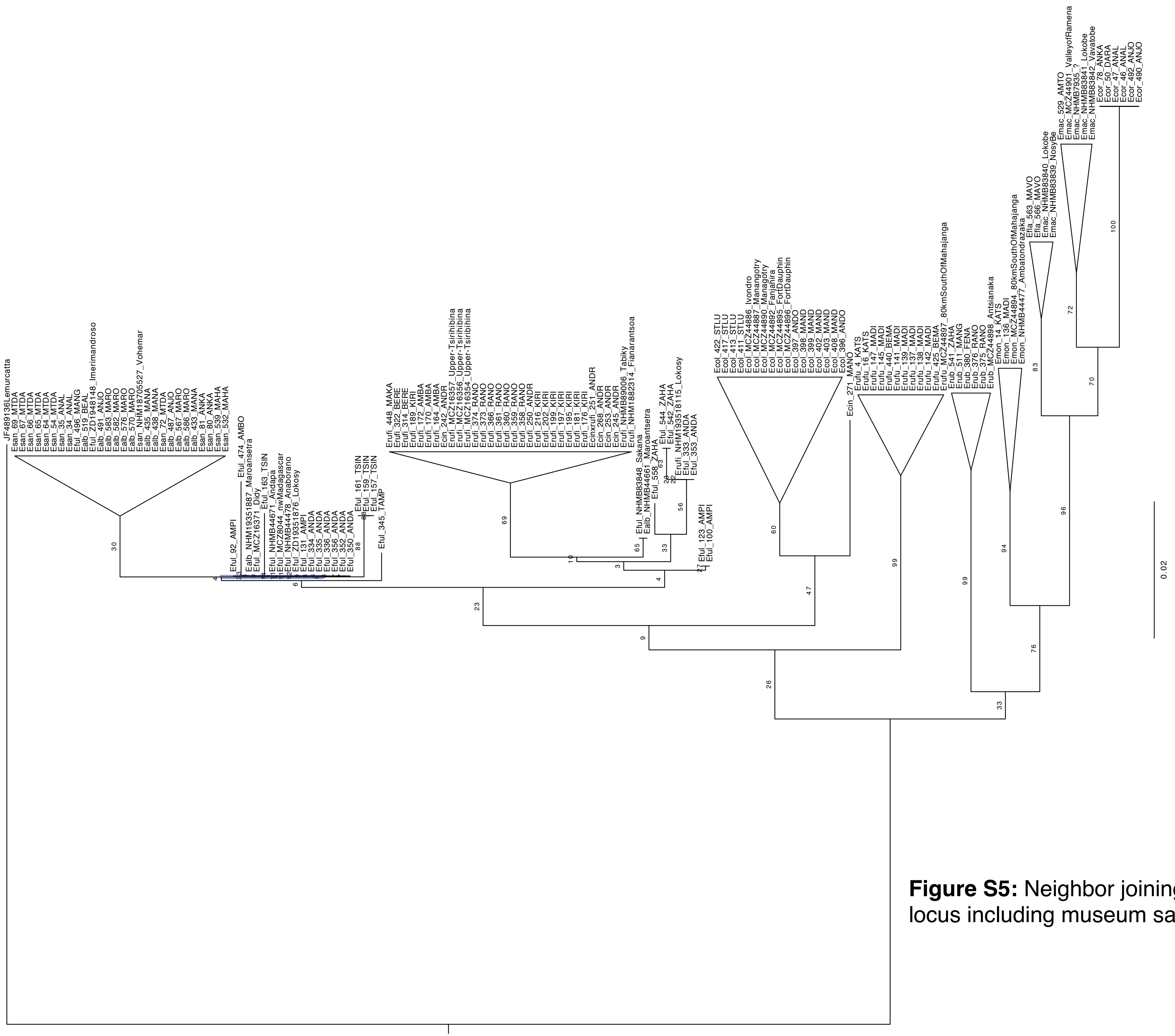

Figure S5: Neighbor joiningtree of the cytb locus including museum samples.

Figure S6a: Genetree of nramp

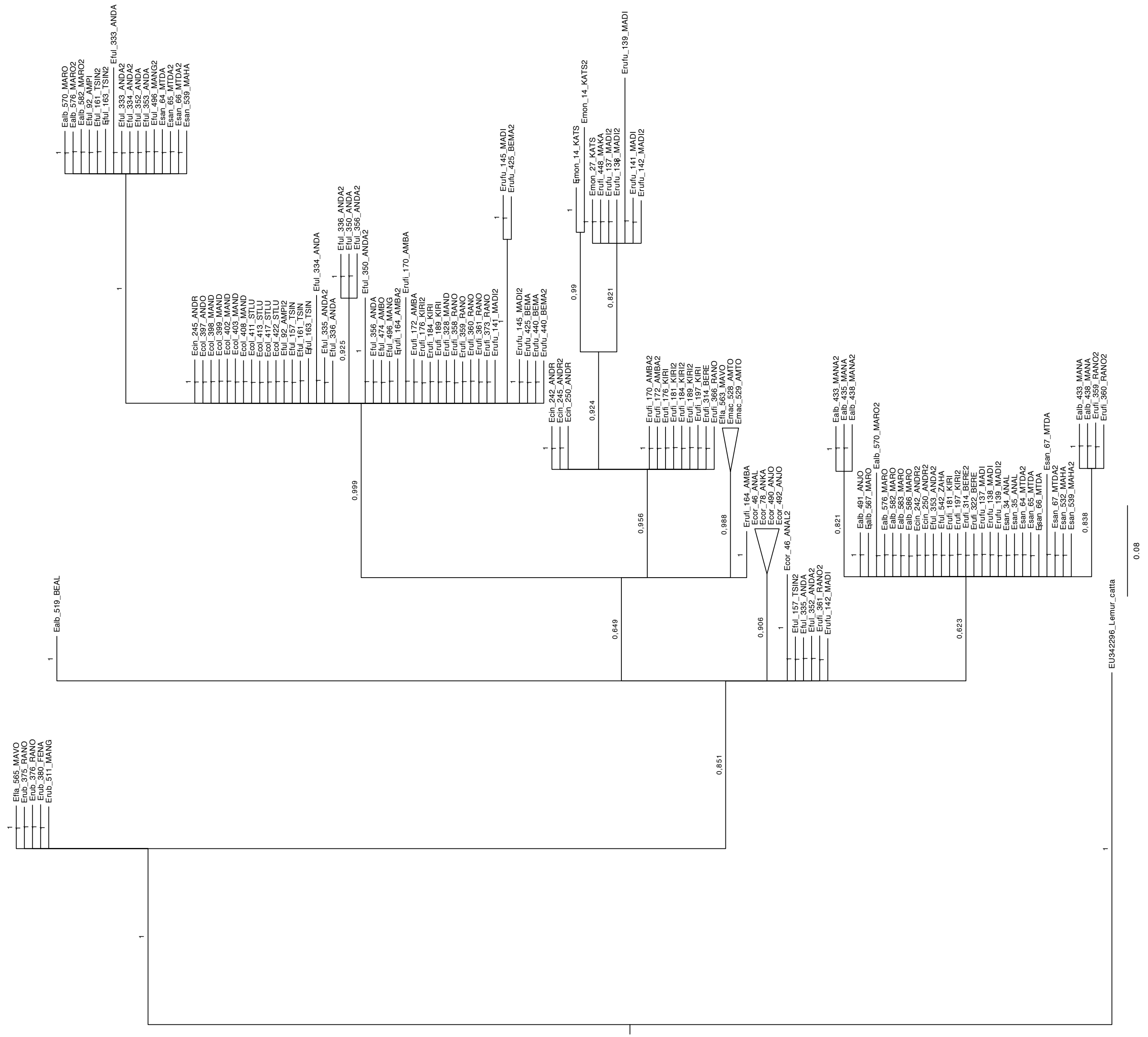

Figure S6b) Genetree of vwf- locus

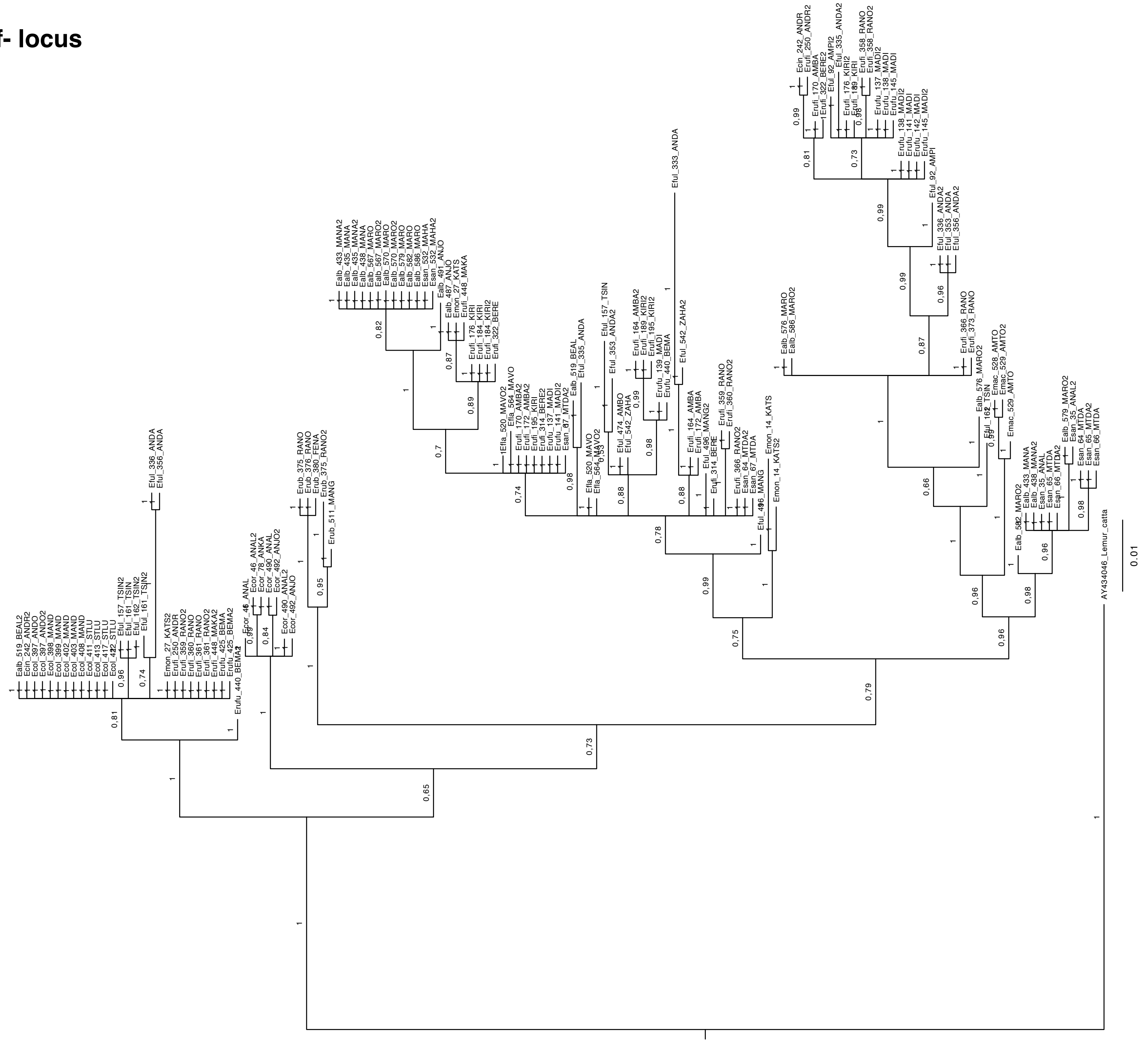

Figure S6c) Gene tree of eno locus

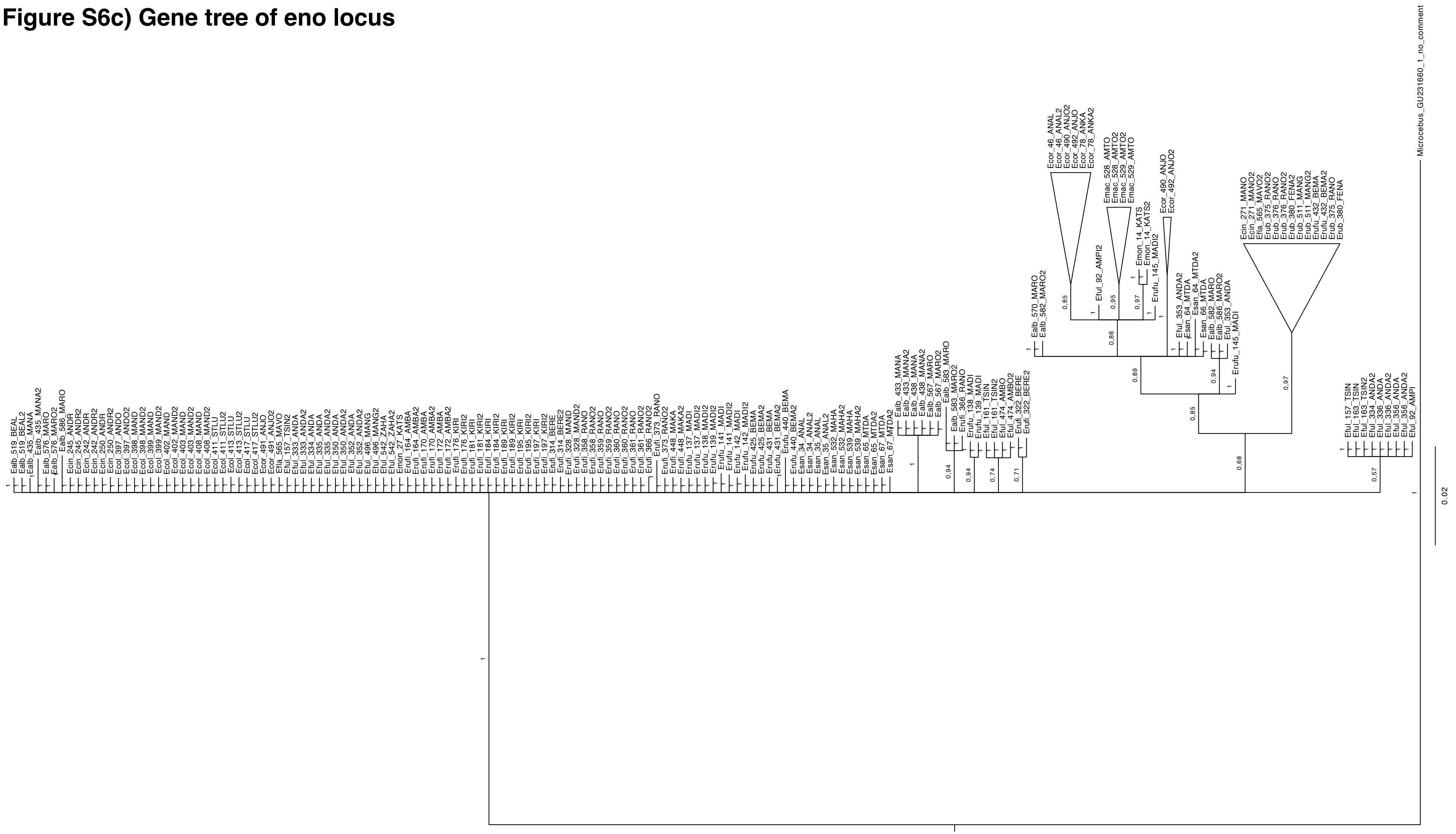

Figure S6a-c): Bayesian gene trees of nuclear loci.
